# IFN-**γ** is essential for alveolar macrophage–driven pulmonary inflammation in macrophage activation syndrome

**DOI:** 10.1172/jci.insight.147593

**Published:** 2021-09-08

**Authors:** Denny K. Gao, Nathan Salomonis, Maggie Henderlight, Christopher Woods, Kairavee Thakkar, Alexei A. Grom, Sherry Thornton, Michael B. Jordan, Kathryn A. Wikenheiser-Brokamp, Grant S. Schulert

**Affiliations:** 1 Division of Rheumatology, Cincinnati Children’s Hospital Medical Center, Cincinnati, Ohio, USA.; 2 Department of Pediatrics, University of Cincinnati College of Medicine, Cincinnati, Ohio, USA.; 3 Division of Biomedical Informatics,; 4 Division of Pathology & Laboratory Medicine, and; 5 Division of Immunobiology, Cincinnati Children’s Hospital Medical Center, Cincinnati, Ohio, USA.; 6 Department of Pathology & Laboratory Medicine, University of Cincinnati College of Medicine, Cincinnati, Ohio, USA.

**Keywords:** Inflammation, Pulmonology, Macrophages, Rheumatology

## Abstract

Macrophage activation syndrome (MAS) is a life-threatening cytokine storm complicating systemic juvenile idiopathic arthritis (SJIA) driven by IFN-γ. SJIA and MAS are also associated with an unexplained emerging inflammatory lung disease (SJIA-LD), with our recent work supporting pulmonary activation of IFN-γ pathways pathologically linking SJIA-LD and MAS. Our objective was to mechanistically define the potentially novel observation of pulmonary inflammation in the TLR9 mouse model of MAS. In acute MAS, lungs exhibit mild but diffuse CD4-predominant, perivascular interstitial inflammation with elevated IFN-γ, IFN-induced chemokines, and alveolar macrophage (AMϕ) expression of IFN-γ–induced genes. Single-cell RNA sequencing confirmed IFN-driven transcriptional changes across lung cell types with myeloid expansion and detection of MAS-specific macrophage populations. Systemic MAS resolution was associated with increased AMϕ and interstitial lymphocytic infiltration. AMϕ transcriptomic analysis confirmed IFN-γ–induced proinflammatory polarization during acute MAS, which switches toward an antiinflammatory phenotype after systemic MAS resolution. Interestingly, recurrent MAS led to increased alveolar inflammation and lung injury, and it reset AMϕ polarization toward a proinflammatory state. Furthermore, in mice bearing macrophages insensitive to IFN-γ, both systemic features of MAS and pulmonary inflammation were attenuated. These findings demonstrate that experimental MAS induces IFN-γ–driven pulmonary inflammation replicating key features of SJIA-LD and provides a model system for testing potentially novel treatments directed toward SJIA-LD.

## Graphical abstract

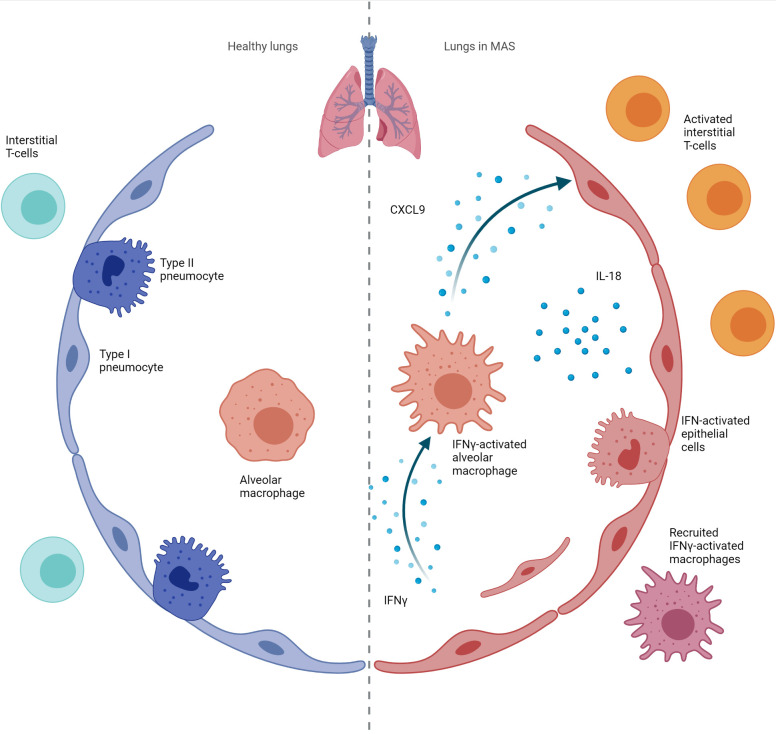

## Introduction

Macrophage activation syndrome (MAS) is a life-threatening “cytokine storm” with clinical similarity to hemophagocytic lymphohistiocytosis (HLH) (1–3). MAS occurs most commonly in the setting of systemic juvenile idiopathic arthritis (SJIA), a distinct subtype of JIA with features of autoinflammation (4). The pathogenesis of familial HLH is dependent on IFN-γ, and neutralization of IFN-γ improves clinical and immunologic features of the disease (5–8). Similarly, recent findings by us and others have found that SJIA-associated MAS is also distinguished by a surge in IFN-γ and IFN-induced chemokines (9, 10).

In contrast to familial HLH, the genetic basis of MAS remains unclear, but several animal models replicate the immunopathology of MAS. The best characterized model was developed by Behrens and colleagues (11), wherein repeated stimulation of mice with the TLR9 agonist CpG induced features reflecting the early or subclinical MAS that affects at least one-third of children with SJIA. These animals also developed peripheral monocytosis sustaining the systemic hyperinflammation, although the precise contribution of myeloid-lineage cells remains undefined (12). Full cytokine storm in this model was dependent on IFN-γ (11, 13, 14).

Along with MAS, an emerging cause of mortality in children with SJIA is chronic lung disease (SJIA-LD) (15), with pulmonary and alveolar inflammation including patchy but extensive lymphoplasmacytic interstitial infiltrates and mixed features of pulmonary alveolar proteinosis (PAP) and endogenous lipoid pneumonia (16). The etiology of SJIA-LD is unknown, but several risk factors and triggers have been proposed. Increasing use of anticytokine therapy for SJIA has been temporally linked to incidence of SJIA-LD (15, 17), with possible allergic reactions as a trigger (17). On the other hand, several large studies have linked SJIA-LD to recurrent episodes of MAS (15–17), and our recent work identified IFN-γ pathway activation in the lungs of children with SJIA-LD (16). Notably, the various causes of PAP are linked through alterations in alveolar macrophage (AMϕ) biology, including impaired functional maturation and polarization (18–21). Together, this raises the intriguing possibility of a connection between MAS, AMϕ biology, and SJIA-LD.

Here, we provide the first direct evidence to our knowledge that mice with MAS exhibit pulmonary inflammation that reflects key features of SJIA-LD, including IFN-γ activation. Single-cell RNA sequencing (scRNA-seq) reveals marked expansion of the monocyte and macrophage compartment in the lungs, including emergence of MAS-specific macrophage populations. Higher levels of pulmonary inflammation were seen after systemic MAS resolution, while recurrent MAS demonstrated a reprograming of AMϕ toward proinflammatory polarization. Finally, mice with macrophages insensitive to IFN-γ (MIIG) showed attenuated lung inflammation. These findings support a model whereby MAS induces IFN-γ–driven pulmonary inflammation and dynamic changes in AMϕ polarization, contributing to the development of SJIA-LD.

## Results

### Pulmonary inflammation in the TLR9 model of MAS.

The best described mouse model of MAS involves repeated administration of the TLR9 agonist CpG, leading to clinical features that closely mimic the “subclinical” MAS observed in nearly one-third of SJIA patients (Supplemental Figure 1; supplemental material available online with this article; https://doi.org/10.1172/jci.insight.147593DS1) (11), including anemia, lymphopenia, thrombocytopenia, massive splenomegaly, and hyperferritinemia (Supplemental Figure 2). MAS was also associated with increased serum cytokine and chemokine levels, consistent with developing cytokine storm. In particular, mice demonstrated increased serum IL-1β, IL-6, IL-10, IL-12p70, IL-17A, TNF, IL-18, and IFN-γ, along with the IFN-induced chemokines CXCL9 and CXCL10 (Supplemental Figure 3).

**Figure 1:**
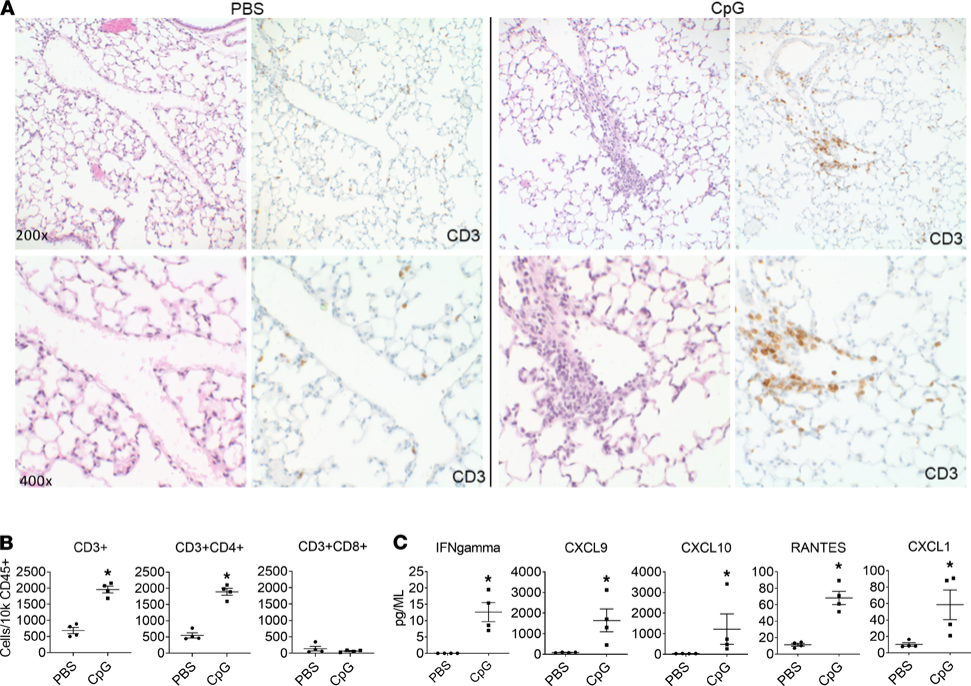
Interstitial pulmonary inflammation in mice during acute MAS. (**A**) Representative histological sections of lung tissue from mice treated with PBS (control) or CpG to induce MAS. Section stained with H&E or with anti-CD3 monoclonal antibody where indicated. Top row, ×200 magnification; bottom row, ×400 magnification. (**B**) Number of CD3^+^, CD3^+^CD4^+^, and CD3^+^CD8^+^ lymphocytes in whole lung tissue from mice treated with PBS or CpG. Data expressed as cells/10,000 CD45^+^ cells. (**C**) Cytokine and chemokine levels from whole lung lysates of mice treated with PBS or CpG. Data are representative of 3 independent experiments (*n* = 4). Data are represented as mean ± SEM. *P < 0.05; Student’s *t* test.

While the above model has become the system of choice for experimental study of MAS, pulmonary involvement in these animals has not been previously reported. Here, we find that lung tissue sections from mice with MAS showed mildly increased mononuclear, lymphocyte-predominant interstitial inflammation that was primarily perivascular but also involving alveolar septa (Figure 1A). In agreement with this, flow cytometry of dissociated lung tissue cells demonstrated a significant increase in CD3^+^ and CD3^+^CD4^+^ T lymphocytes in mice with acute MAS compared with control (PBS-treated) mice (Figure 1B). Homogenized whole lung tissue in acute MAS also showed significantly increased levels of IFN-γ and the IFN-induced chemokines CXCL9 and CXCL10, as well as RANTES and CXCL1 (Figure 1C). Interestingly, other proinflammatory cytokines that were significantly elevated in serum (including IL-1β, IL-6, and IL-10) were unchanged in lung tissue (Supplemental Figure 4A), suggesting that increases in IFN-γ and IFN-induced chemokines may not simply reflect systemic levels or contamination from pulmonary vasculature. We provide the first direct demonstration to our knowledge that mice with acute MAS have a mild lymphocyte-predominant pulmonary interstitial inflammation associated with increased IFN-γ and IFN-induced chemokines.

### AMϕ activation in acute MAS.

**Figure 2:**
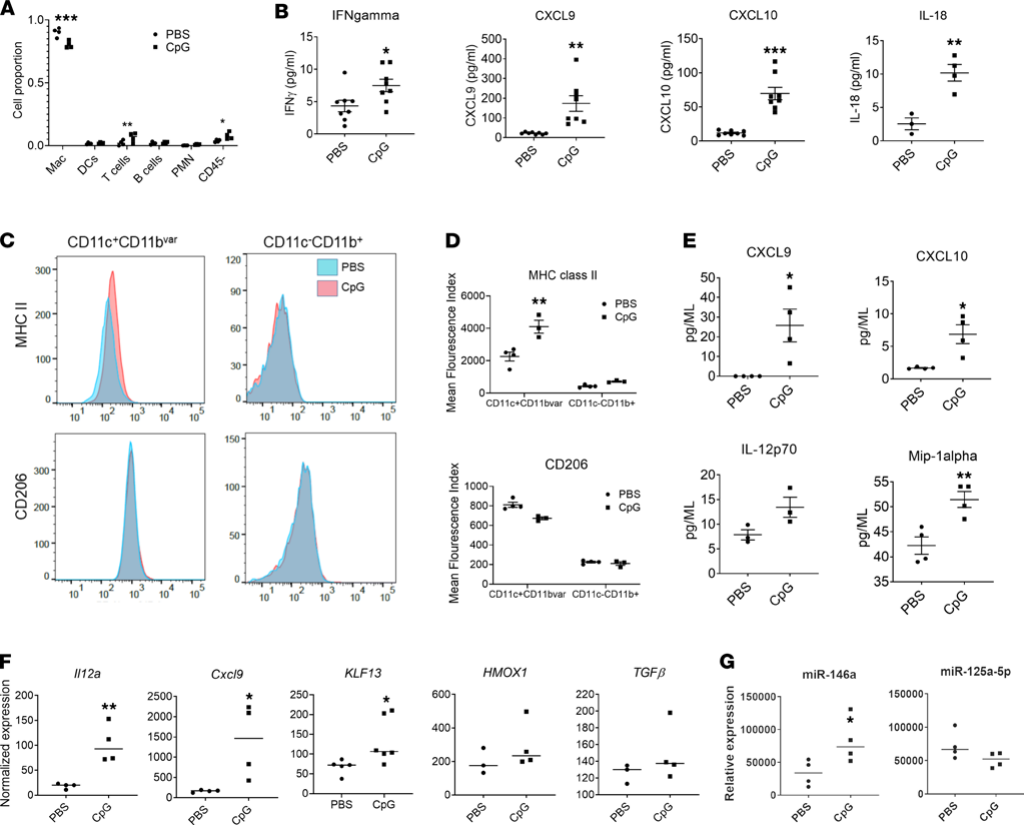
AMϕ demonstrate features of IFN-γ–mediated activation during acute MAS. (**A**) Relative proportion of cell types in BAL determined by flow cytometry. (**B**) BAL fluid cytokine and chemokine levels. (**C** and **D**) MHCII and CD206 surface expression in lung macrophage populations as determined by flow cytometry. (**C**) Representative histograms. (**D**) Mean fluorescence index. (**E**) Cytokine and chemokine release by AMϕ from control mice or mice treated with CpG. (**F**) AMϕ gene expression as determined by qPCR. (**G**) AMϕ microRNA expression. Data are representative of 3 independent experiments (*n* = 4 or 8). Data are represented as mean ± SEM. *P < 0.05; **P < 0.01; ***P < 0.001; Student’s *t* test.

Given the histology seen in SJIA-LD, and the link between PAP and AMϕ dysfunction, we performed bronchoalveolar lavage (BAL) on mice with acute MAS to assess for signs of airway inflammation. While mice with CpG-induced MAS did not show increased total alveolar cells (data not shown), there was a small but significant decrease in the AMϕ proportion and an increase in T cells and CD45^–^ epithelial cells during acute MAS (Figure 2A). In addition, BAL fluid showed a significant increase in IL-18, IFN-γ, CXCL9, and CXCL10 (Figure 2B), similar to that found in SJIA-LD patients (16). In contrast, levels of other cytokines including IL-1β, IL-6, IL-10, IL-12, and TNF were unchanged (Supplemental Figure 4B). We also found that CD11c^+^CD11b^variable^CD64^+^ (“variable” hereafter written as “var”) AMϕ had significantly increased surface expression of MHC class II (MHCII) markers, reflecting classical activation, without any significant changes in M2 markers such as CD206 (Figure 2, C and D). In contrast, there were no significant changes in surface marker expression in CD11c^–^CD11b^+^ macrophages. AMϕ also showed significant upregulation in IFN-γ–induced, proinflammatory genes including *IL12A* and *CXCL9*, without changes in other polarization markers such as *HMOX1* and *TGFB* (Figure 2F). AMϕ also showed significant upregulation of *KLF13* mRNA, which is essential for M1 polarization in mice (22). MicroRNAs are increasingly recognized as key regulators of macrophage polarization (23). Here, we find increased expression of the proinflammatory miR-146a, but no significant changes in the key regulatory miR–125a-5p, which directly targets *KLF13* (Figure 2G). Finally, we found that AMϕ from mice with acute MAS secreted significantly increased levels of CXCL9 and CXCL10, along with IL-12p70 and Mip-1α, when cultured ex vivo compared with cells from control mice (Figure 2E). Taken together, we find that, during MAS, AMϕ demonstrate changes in surface MHCII and gene expression, reflecting proinflammatory polarization and IFN-γ activation, and they secrete IFN-induced chemokines into BAL fluid. Notably, the phenotype of AMϕ in MAS does not simply reflect that of mouse peripheral blood monocytes, which have distinct gene expression signatures including failure to increase *IL12A* or *KLF13* and decreased expression of *TGFB* and *IL10* (Supplemental Figure 5). These divergent transcriptional responses may reflect differential and tissue-specific effects of IFN-γ, along with inherent properties of AMϕ, and they support distinct effects of MAS in the lungs.

### Broad IFN-induced transcriptional responses and myeloid expansion and diversification revealed with scRNA-seq.

**Figure 3:**
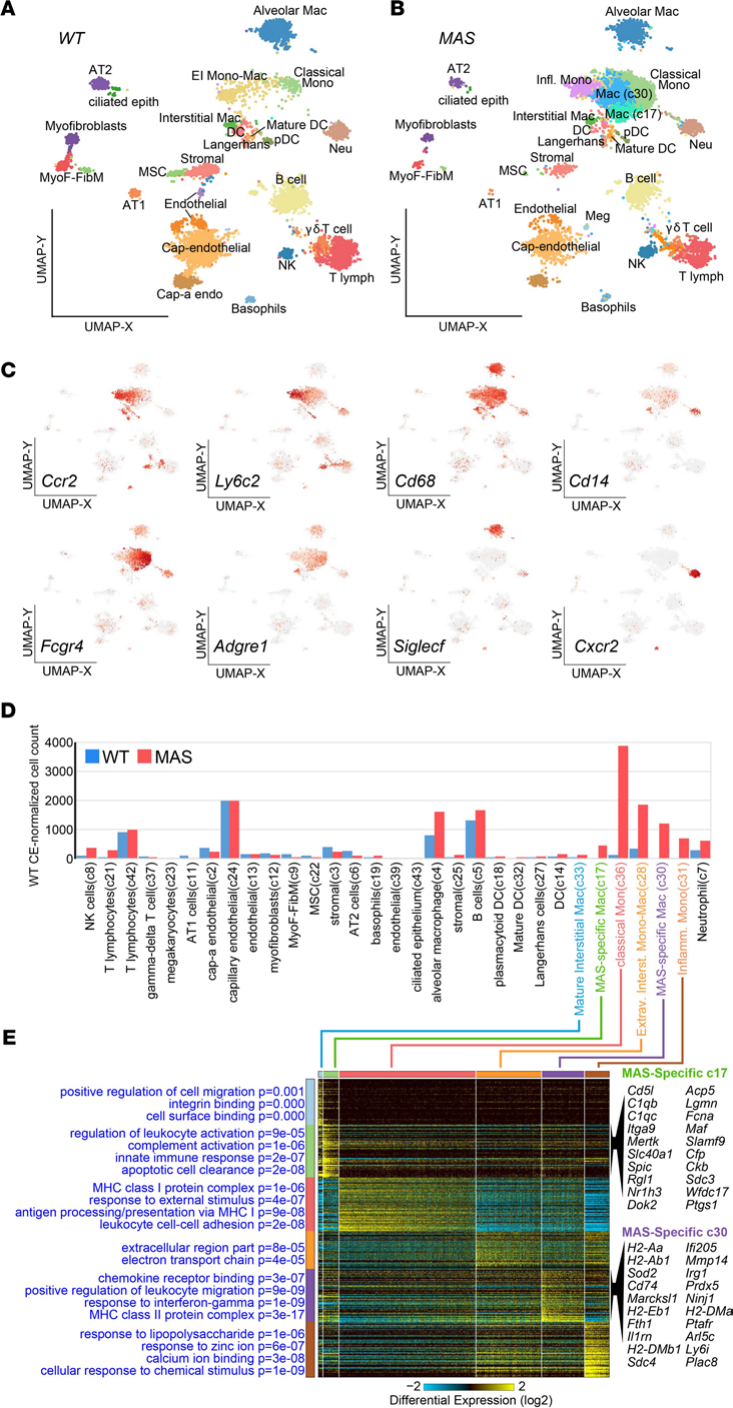
Distinct immune single-cell populations infiltrate the lung in MAS. (**A** and **B**) UMAP projection of single-cell populations defined by the software ICGS2 following joint-analysis of WT (**A**) and MAS (**B**) lung. (**C**) UMAP projection of gene expression for selected markers genes (gray, no expression; red, high). (**D**) The percent of each cell population is shown, normalizing the number of cells in the MAS to WT (PBS) capillary endothelial (c24), which should have similar frequencies in WT and MAS captures. (**E**) Heatmap of the top monocyte and macrophage cluster markers (top 60) from the MAS scRNA-seq using the MarkerFinder algorithm. Top-enriched GO terms and associated enrichment p-values (GO-Elite) are indicated on the left of the heatmap. The top 20 marker genes for each MAS-specific macrophage cluster are shown to the right of the corresponding heatmap clusters.

To better identify these cellular and molecular impacts of MAS across lung cell populations, we performed scRNA-seq using the 10X Genomics platform to interrogate ~15,000 single cells from WT (PBS-treated) and MAS lung tissue (Supplemental Table 1). Using the unsupervised single-cell population discovery and annotation pipeline ICGS2, we identified 33 transcriptionally unique cell clusters in the combined WT and MAS captures (Figure 3, A and B, and Supplemental Tables 2 and 3). Two of these clusters were excluded based on their broad distribution within the produced uniform manifold approximation and projection (UMAP) graph and lack of population specific protein-coding marker genes, which were predicted as unknown cell types by ICGS2. We aligned the cell-type prediction names to the literature and confirmed their specificity based on the expression of previously defined markers (Figure 3C). As expected, distinct monocytic, macrophage, neutrophil, and T and B cell subsets were enriched in MAS, with less frequent detection of epithelial and endothelial cell populations. When normalized to the most frequently detected endothelial cell population in both captures, we found the most significant increases during MAS in classical monocytes (the dominant cell population in MAS), extravasating interstitial mono-mac cells, and inflammatory monocytes, as well as 2 MAS-specific macrophage clusters (c17 and c30) absent in WT lung tissue (Figure 3D). Genes uniquely expressed in cluster c17 macrophages were most enriched in pathways for complement activation and apoptotic clearance, and they expressed markers of recruited airway macrophages (24) including *CD14*, *MAFB*, and *CCR5* (Figure 3E, Supplemental Figure 6, and Supplemental Tables 4 and 5). Compared with classical monocytes (c36), c17 also downregulated *NFKB1*, an NF-κB repressor (25), and upregulated many of its targets including *IL18* expression. In contrast, uniquely expressed genes in macrophage cluster c30 were most enriched in response to IFN-γ, leukocyte migration, and chemokine receptor binding pathways, including *CXCL9*, *CXCL10*, and *TNF* (Figure 3E, Supplemental Figure 6, and Supplemental Tables 4 and 5).

**Figure 4:**
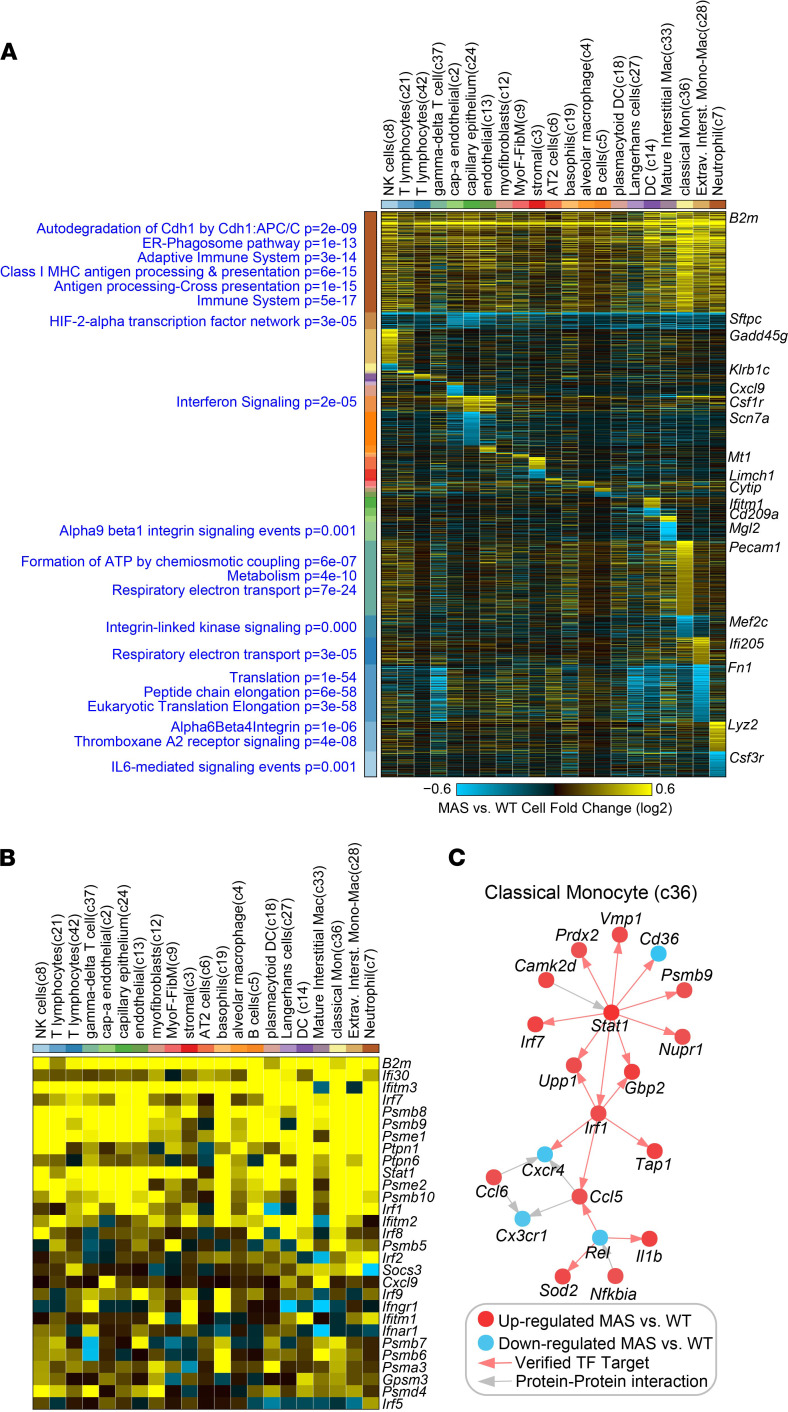
IFN signaling networks are broadly impacted in distinct MAS cell populations. (**A**) Heatmap of MAS versus WT fold-changes (log_2_) for all differentially expressed genes comparing the same cell populations detected in WT (PBS) and MAS scRNA-seq by the software cellHarmony. The heatmap is broken down according to broad and cell type–specific patterns of MAS-induced gene changes, using the cellHarmony default display. Enriched PathwayCommons gene sets are displayed to the left of the heatmap, while the associated GO-Elite enrichment *P* values (blue) and example genes from each cellHarmony gene module are shown to the right of the heatmap. (**B**) Filtered version of the heatmap from **A**, for IFN signaling–associated genes (ToppFun). (**C**) The predicted gene regulatory network for MAS versus WT genes in the classical monocyte cell population (cellHarmony).

To more broadly assess gene expression differences in each pair of MAS and WT cell populations, we performed an exhaustive comparison analysis using the cellHarmony workflow with the cell labels assigned from ICGS2. This analysis highlighted close to 1900 confidently differentially expressed genes (DEGs) across 21 of 31 compared cell populations, in which sufficient cell numbers were present for comparison (Figure 4A and Supplemental Table 6). Most notably, genes associated with the adaptive immune system were highly, broadly, and consistently upregulated across the majority of cell populations in MAS, whereas hypoxia genes were consistently downregulated. Among genes broadly upregulated across cell populations were those associated with IFN signaling, including a number proteasomal components in addition to IFN receptors (Figure 4B). In addition, highly specific gene programs were impacted in each of the monocyte and macrophage cell populations, with the largest transcriptomic impact in classical monocytes (Figure 4A). While classical monocyte upregulated genes were selectively enriched in energy metabolism and TCA genes, cellHarmony predicted regulation of a core Stat1 transcriptional network in these cells, in combination with Irf1 and Rel responsive transcripts, by virtue of prior experimentally evidenced direct targets (Figure 4C). Finally, T cell populations (c42) showed gene signatures reflecting activation including IL-2–mediated signaling events (adjusted *P* = 0.001). Taken together, these findings demonstrate that experimental MAS induced a broad IFN-induced transcriptional response throughout the lung, along with massive expansion and emergence of potentially novel monocyte and macrophage populations.

### Persistent pulmonary inflammation, with a shift in AMϕ phenotype after systemic MAS resolution.

Most patients with SJIA-LD develop pulmonary complications, not at systemic disease onset, but in the subsequent months (16, 17). Therefore, we have further developed this TLR9 model system by, for the first time to our knowledge, characterizing the resolution phase of MAS (Supplemental Figure 1). We found that, 3 weeks after MAS induction, CpG-treated mice largely resolved their systemic cytokine storm including cytopenias (Supplemental Figure 7A) and had markedly reduced spleen size compared with acute MAS (though still slightly larger than control mice; Supplemental Figure 7, B and C). In addition, the serum cytokine profile including IFN-γ and IL-6 had largely normalized at this later time point, with the notable exception of persistent IL-18 elevation (Supplemental Figure 7D). Interestingly, IL-18 remains chronically elevated in patients with SJIA for many months, despite achieving clinically inactive disease (26).

**Figure 5:**
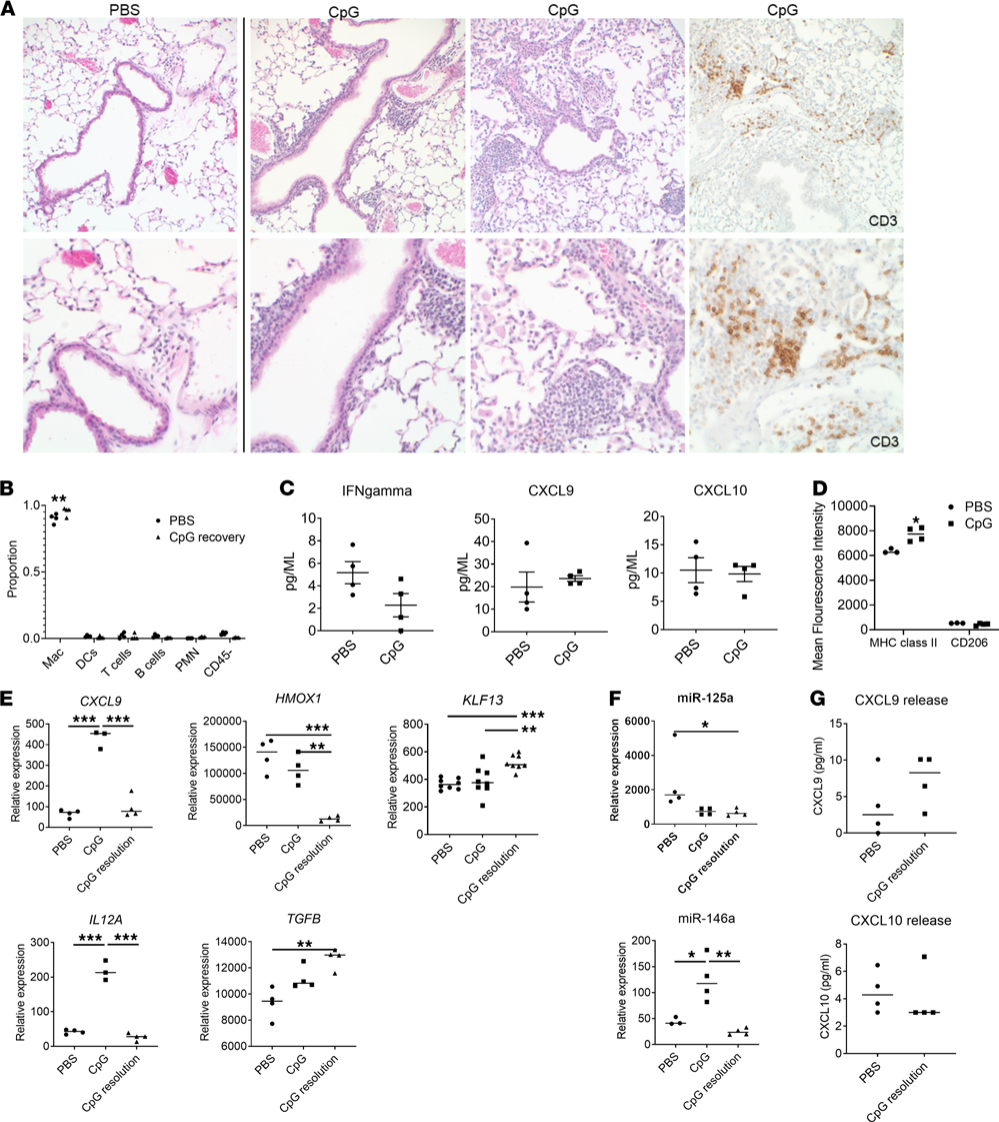
Persistent interstitial inflammation and AMϕ changes after systemic MAS resolution. (**A**) Representative histological sections of lung tissue from mice treated with PBS (control) or CpG to induce MAS, after 3-week recovery. Sections stained with H&E or with anti-CD3 monoclonal antibody where indicated. Top row, ×200 magnification; bottom row, ×400 magnification. (**B** and **C**) BAL fluid relative cell proportions (**B**), and cytokine and chemokine levels (**C**), after systemic MAS resolution. (**D**) MHCII and CD206 surface expression in CD11c^+^CD11b^var^ lung macrophages. (**E** and **F**) AMϕ gene expression as determined by qPCR (**E**) and microRNA expression (**F**) in mice with acute MAS (CpG) and MAS resolution (CpG resolution). (**G**) Chemokine release from cultured AMϕ from control and CpG-treated mice after systemic MAS resolution. Data are representative of 3 independent experiments (*n* = 4–8). Data are represented as mean ± SEM. *P < 0.05; **P < 0.01; ***P < 0.001; Student’s *t* test (PBS versus CpG) or ANOVA with follow-up Dunnett’s multiple-comparison test.

Mice after systemic MAS resolution continue to have pulmonary interstitial inflammation (Figure 5). Histologic examination demonstrated lymphocyte-predominant, chronic mononuclear perivascular and peribronchiolar infiltrates, findings that were variable among mice but diffuse involving multiple lung lobes with focal lymphocyte aggregates (Figure 5A). Analysis of BAL fluid after systemic MAS resolution revealed a modest but significant increase in total number of alveolar cells as well as the proportion of AMϕ, not seen during acute MAS, providing evidence of disease progression (Figure 5B). In order to quantify the scattered interstitial inflammation, we developed an Aperio-based staining algorithm on digitized slides to quantify positively stained cells (Supplemental Figure 8). Mice with resolving MAS showed a nearly 50% increase in the number of CD3^+^ cells/mm^2^ of lung compared with untreated control mice (average 47.5 versus 32.2 positive cells/mm^2^, respectively). Consistent with the serum cytokines, BAL chemokine levels had normalized by this stage (Figure 5C), and cultured AMϕ did not secrete significant levels of chemokines (Figure 5G). However, CD11c^+^CD11b^var^CD64^+^ AMϕ continued to have a small but significant increase in MHCII expression, without any difference in CD206 expression (Figure 5D). Targeted gene expression analysis demonstrated a shift in AMϕ polarization phenotypes during MAS recovery. While expression of IFN-induced genes *CXCL9* and *IL12A*, as well as the M1-associated miR-146a, had normalized, other polarization markers were significantly altered, evident by the downregulation of *HMOX1* and upregulation of *TGFB* (Figure 5, E–G). In addition, miR–125a-5p levels were significantly reduced, while its target *KLF13* remained elevated. In total, we found that, during the resolution phase of TLR9-induced MAS, mice demonstrate (a) persistent interstitial inflammation, (b) increased AMϕ, and (c) further alterations in AMϕ gene and microRNA expression, suggesting a shift in macrophage polarization.

### Transcriptional profiling of AMϕ shows shift in polarization phenotypes between acute MAS and resolution.

**Figure 6:**
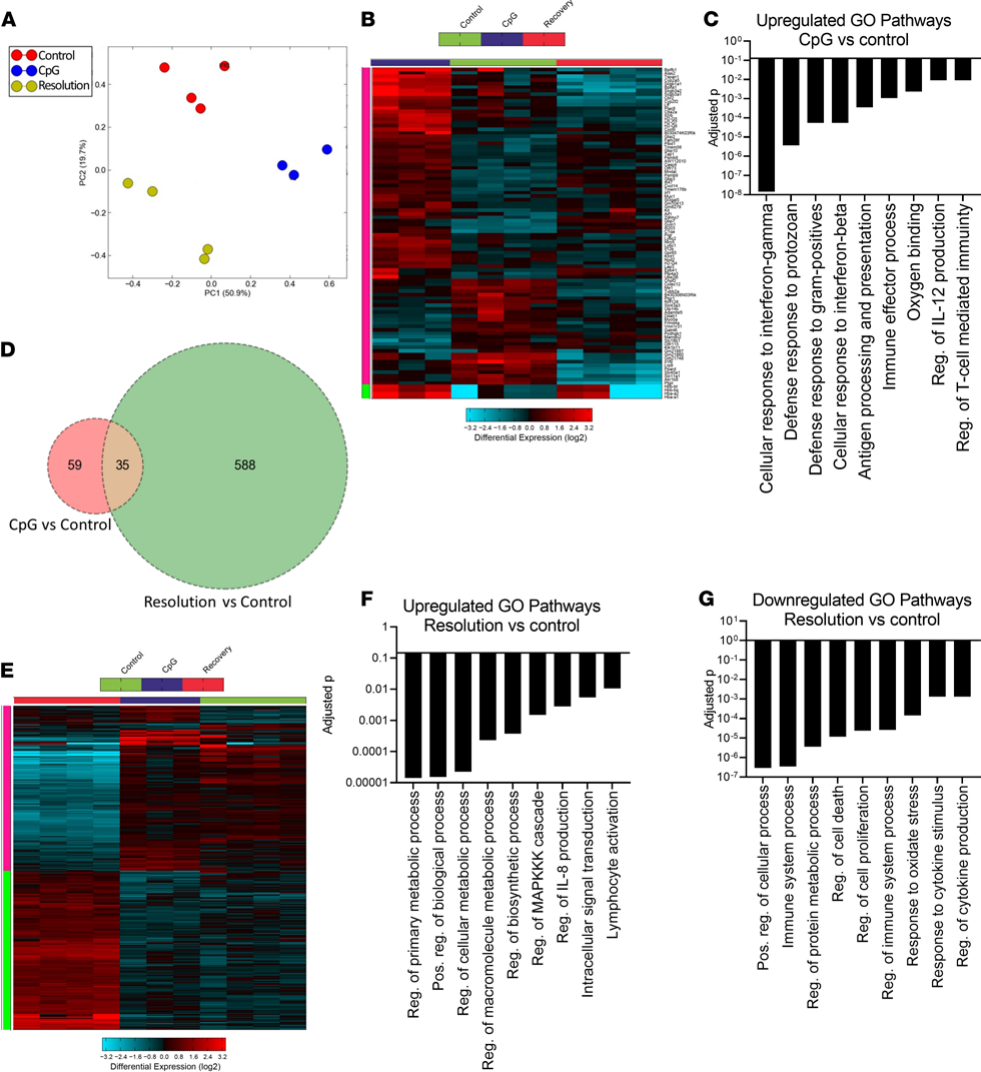
Gene expression analysis of AMϕ in acute and resolving MAS. (**A**) Principal component analysis. (**B**) Hierarchical clustering of differentially expressed genes (DEG) between acute MAS and control macrophages (fold change > 2, *P* < 0.05). (**C**) Most significantly enriched GO pathways of genes upregulated in acute MAS AMϕ versus control. (**D**) Venn diagram of differentially regulated genes in acute and resolving MAS versus control AMϕ. (**E**) Hierarchical clustering of DEG between systemic MAS resolution and control. (**F**) Most significantly enriched GO pathways of genes upregulated in resolving MAS AMϕ versus control. (**G**) Most significantly enriched GO pathways of genes downregulated in resolving MAS AMϕ versus control.

Genome-wide transcriptional profiles using microarray were generated of adherence-purified AMϕ from control mice (PBS-treated) or mice with acute MAS or after systemic MAS resolution (Supplemental Figure 1). Principal component analysis showed that samples from the 3 treatment groups were clearly distinct (Figure 6A). Comparing acute MAS to control AMϕ, we identified 94 DEGs (fold change > 2, *P* < 0.05) including 64 upregulated and 30 downregulated genes (Figure 6B and Supplemental Tables 7 and 8). Pathway analysis on these DEGs identified cellular response to IFN-γ (Gene Ontology [GO] 0071346) as the most significantly enriched pathway among upregulated genes (*Z* score 25.0, adjusted *P* = 1.47 × 10^–8^). Other enriched GO pathways are shown in Figure 6C and support a proinflammatory phenotype, while downregulated genes did not show any significantly enriched GO pathways.

Interestingly, transcriptional profiles of AMϕ in the resolution phase of MAS displayed much more extensive transcriptional changes than during acute MAS, with 316 upregulated and 307 downregulated genes compared with control AMϕ (Figure 6, D and E, and Supplemental Table 9). The most enriched GO pathways of upregulated genes in resolution phase AMϕ included those involved in regulation of metabolic processes (GO:0080090, *Z* score 6.6, adjusted *P* = 1.5 × 10^–5^; Figure 6F). Specific upregulated genes include several transcription factors of the Kruppel-like factor (KLF) family (Supplemental Table 10), including *KLF13*, with key roles in cellular metabolism and macrophage polarization, including regulatory and antiinflammatory phenotypes (27–29). There was no evidence of an IFN-induced signature in resolution phase AMϕ. On the other hand, upregulated genes did include a STAT6-regulated cluster (*Z* score 5.0, adjusted *P* = 0.03), which is induced by cytokines that drive M2 polarization (Supplemental Figure 8) (30). In contrast, downregulated gene pathways in AMϕ during MAS resolution encompassed cellular and immune processes and responses to cytokine stimulation (Figure 6G), including those regulated by IFN-activated STAT1 (*Z* score 4.1, adjusted *P* = 0.05) and by Bach1 (*Z* score 6.1, adjusted *P* = 0.01) (Supplemental Figure 9). Bach1 is notable, as loss of this activity in mice may exacerbate certain forms of PAP (20). Taken together, we find that AMϕ — after systemic MAS resolution — exhibit large-scale transcriptional changes impacting inflammatory and metabolic pathways toward a more antiinflammatory and regulatory polarization state.

### Augmented pulmonary inflammation and lung injury, and AMϕ reprograming in recurrent MAS.

**Figure 7:**
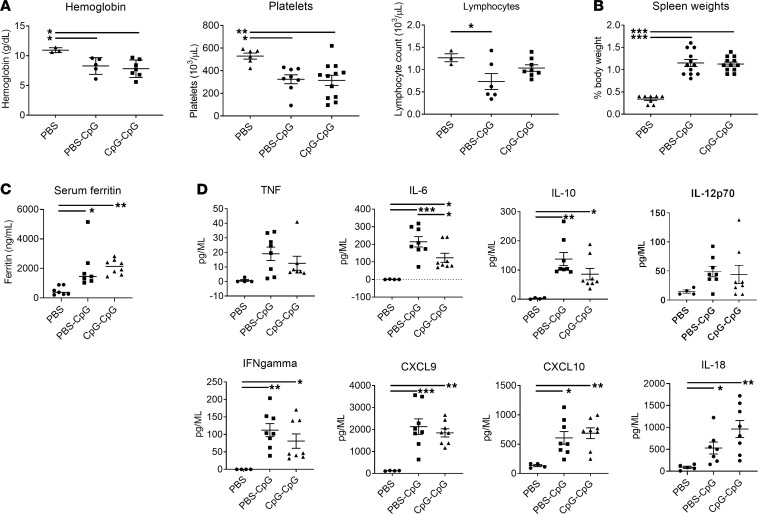
Systemic features in mice with recurrent MAS. (**A**–**C**) Hemoglobin, platelet, and absolute lymphocyte counts (**A**), spleen weights (**B**), and serum ferritin (**C**) in control mice or mice with acute MAS (PBS-CpG) or recurrent MAS (CpG-CpG). (**D**) Serum cytokine levels as determined by luminex assay. Data are representative of 3 independent experiments (*n* = 4 or 8). Data are represented as mean ± SEM. *P < 0.05; **P < 0.01; ***P < 0.001; ANOVA with follow-up Dunnett’s multiple-comparison test.

Many patients with SJIA-LD suffer from repeated episodes of overt or subclinical MAS. Therefore, we have treated mice with either PBS or CpG (round 1), followed by retreatment with CpG (round 2), yielding controls (PBS) as well as mice with 1 (PBS-CpG) or 2 (CpG-CpG) episodes of MAS (Supplemental Figure 1). Overall, most systemic features of MAS were similar between PBS-CpG and CpG-CpG, including anemia and thrombocytopenia (Figure 7A), splenomegaly (Figure 7B), and hyperferritinemia (Figure 7C), although mice with recurrent MAS did not show significant lymphopenia (Figure 7A). While serum cytokine profiles showed largely similar increases between PBS-CpG and CpG-CpG (Figure 7D), serum IL-18 levels showed a trend toward increased levels in mice with recurrent MAS (*P* = 0.09), in agreement with elevated IL-18 in patients with a history of MAS (26).

**Figure 8:**
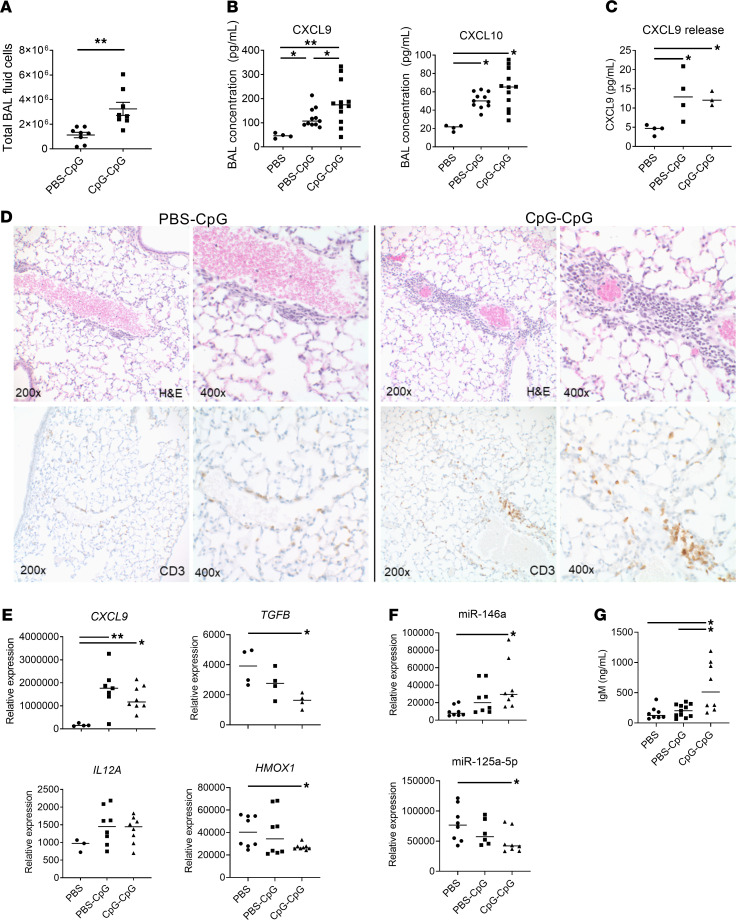
Enhanced pulmonary inflammation and lung injury with repeated episodes of MAS. (**A**) Total BAL fluid cell counts in mice with 1 (PBS-CpG) or 2 (CpG-CpG) episodes of MAS. (**B**) BAL fluid chemokine levels in control mice (PBS) or those with 1 (PBS-CpG) or 2 (CpG-CpG) episodes of MAS. (**C**) In vitro CXCL9 release from AMϕ isolated from control mice (PBS) or those with 1 (PBS-CpG) or 2 (CpG-CpG) episodes of MAS. (**D**) Representative histological sections of lung tissue with 1 (left) or 2 (right) episodes of MAS. Sections were stained with H&E (top) or anti-CD3 (bottom). (**E** and **F**) AMϕ gene expression as determined by qPCR (**E**) and microRNA expression (**F**) in control mice (PBS) or those with 1 (PBS-CpG) or 2 (CpG-CpG) episodes of MAS. (**G**) BAL IgM concentration as determined by ELISA. Data are representative of 3 independent experiments (*n* = 4 or 8). Data are represented as mean ± SEM. *P < 0.05; **P < 0.01; ***P < 0.001; ANOVA with follow-up Dunnett’s multiple-comparison test.

We next examined whether recurrent MAS altered features of pulmonary inflammation and AMϕ polarization. Mice with recurrent MAS continued to show significantly higher numbers of AMϕ (Figure 8A) and significantly higher BAL levels of CXCL9 and CXCL10 compared with those with a single MAS episode (Figure 8B). This is likely related to the enhanced number of AMϕ, as ex vivo chemokine release was similar between AMϕ from PBS-CpG and CpG-CpG mice (Figure 8C). Histologically, mice with recurrent MAS demonstrated similar findings of increased chronic lymphocytic perivascular infiltrates; however, inflammation was more extensive and more diffuse throughout lung lobes than in acute MAS, including presence of focal aggregates rarely seen after a single acute treatment (Figure 8D). Isolated AMϕ in recurrent MAS showed a reprograming from the antiinflammatory phenotype seen after systemic MAS resolution, toward a similar proinflammatory phenotype seen in acute MAS with activation of IFN-γ–induced genes such as *CXCL9* and *IL12A* (Figure 8E); it also showed a decreases in *TGFB* and *HMOX1* (Figure 8E), as well as more pronounced changes in miR-146a and miR-125a-5p (Figure 8F).

Finally, we quantified levels of IgM protein in BAL fluid as a marker of epithelial permeability and lung injury (31). While BAL protein concentration was unchanged from control in acute MAS, recurrent MAS showed a significant increase in IgM concentration (Figure 8G). Overall, mice with recurrent TLR9-induced MAS showed more pronounced lung inflammation and lung injury than seen in acute MAS, as well as a reprograming of AMϕ phenotypes from resolution toward proinflammatory polarization.

### Blockade of IFN-γ limits features of pulmonary inflammation.

**Figure 9:**
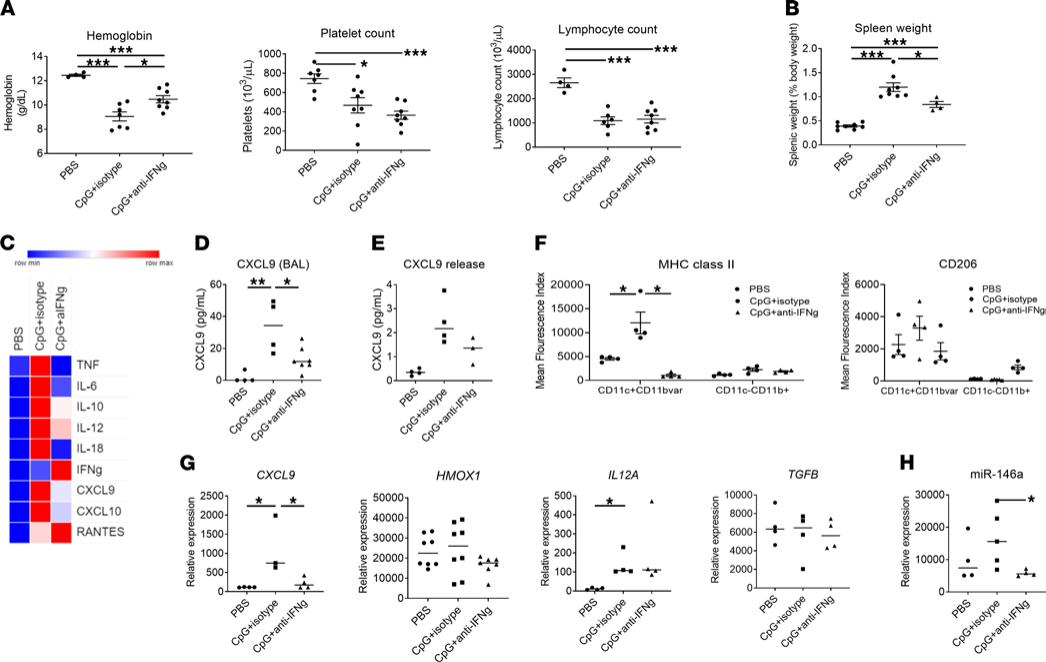
Systemic and pulmonary inflammation in mice with MAS and IFN-γ blockade. (**A** and **B**) CpG + isotype control and CpG + anti–IFN-γ–treated mice demonstrate mild anemia (left), thrombocytopenia (center), and lymphopenia (right) (**A**), as well as splenomegaly (**B**), compared with mice treated with PBS alone. (**C**) Cytokine levels as determined by luminex, except for IL-18, which was determined by specific ELISA. Data are mean of 4 mice per condition, normalized for each analyte. (**D**) BAL fluid CXCL9 levels. (**E**) CXCL9 release by AMϕ. (**F**) AMϕ MHCII and CD206 surface expression as determined by flow cytometry. (**G** and **H**) AMϕ gene (**G**) and microRNA (**H**) expression as determined by qPCR. Data are representative of 3 independent experiments (*n* = 4–8). Data are represented as mean ± SEM. *P < 0.05; **P < 0.01; ***P < 0.001; ANOVA with follow-up Dunnett’s multiple-comparison test.

Previous work has shown that signs of IFN-γ pathway activation are also observed in the lungs of children with SJIA-LD (16). Since blockade of IFN-γ reduces the systemic cytokine storm in the TLR9 model of MAS (11, 13, 14), we investigated whether IFN-γ blockade could similarly limit pulmonary and airway inflammation. Administration of IFN-γ neutralizing antibodies concomitant with CpG treatment as described (11) significantly reduced some systemic features of MAS — most notably, anemia and splenomegaly (Figure 9, A and B). IFN-γ blockade also attenuated the elevation of several serum cytokines and chemokines, including IL-6, IL-10, TNF, CXCL9, and CXCL10, although others such as IL-12 and RANTES appeared relatively unaffected, possibly reflecting the incomplete nature of systemic IFN-γ blockade using the monoclonal antibody (Figure 9C).

In the lungs, IFN-γ blockade significantly reduced BAL chemokine levels, including CXCL9 levels (Figure 9D). This was accompanied by a reduced ability of AMϕ to secrete CXCL9 ex vivo (Figure 9E). In addition, IFN-γ blockade reduced expression of MHCII activation markers specifically on CD11c^+^CD11b^var^CD64^+^ AMϕ (Figure 9F). IFN-γ blockade also reduced expression of *CXCL9* by AMϕ; however, *IL12A* and miR-146a expression were largely unchanged (Figure 9, G and H). However, there were no clear histological differences in the lungs when mice were given anti–IFN-γ antibody concomitant with CpG (data not shown). These findings show that AMϕ activation in the TLR9 model of MAS is at least partially IFN-γ dependent.

### Macrophage lineage response to IFN-γ is essential for full systemic cytokine storm and alveolar inflammation.

**Figure 10:**
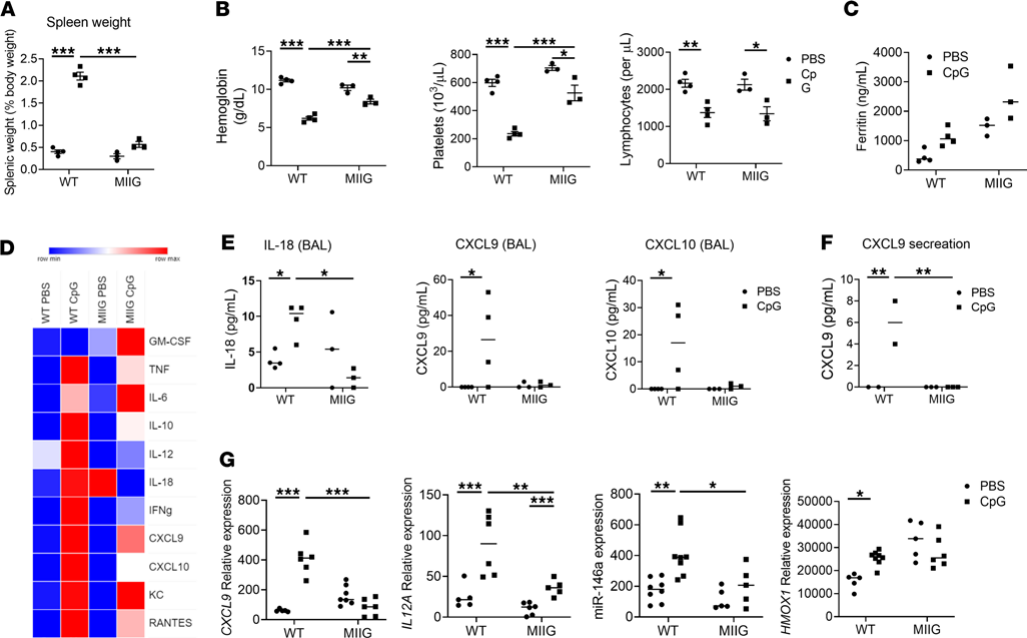
Macrophage sensitivity to IFN-γ is essential for full systemic cytokine storm and alveolar inflammation. (**A**) Spleen weights in WT or MIIG mice treated with PBS or CpG. (**B**) Hemoglobin, platelet, and lymphocyte counts in WT or MIIG mice treated with PBS or CpG. (**C**) Serum ferritin level. (**D**) Cytokine levels as determined by luminex, except for IL-18, which was determined by specific ELISA. Data are mean of 3 mice per condition, normalized for each analyte. (**E**) Concentration of IL-18, CXCL9, and CXCL10 in BAL fluid. (**F**) Concentration of CXCL9 secreted by ex vivo cultured AMϕ. (**G**) AMϕ gene expression as determined by qPCR. *n* = 3 mice per condition. Data are represented as mean ± SEM. *P < 0.05; **P < 0.01; ***P < 0.001; ANOVA with follow-up Dunnett’s multiple-comparison test.

To further define the role of systemic and AMϕ responses to IFN-γ in MAS, we utilized MIIG mice (32), expressing a dominant-negative *IFNGR1* in CD68^+^ cells, with tissue macrophages including AMϕ unresponsive to IFN-γ stimulation (32). Compared with WT, MIIG mice showed significant reductions in key clinical features of MAS, including splenomegaly, anemia, and thrombocytopenia (Figure 10, A and B), while others such as lymphopenia and hyperferritinemia were largely unchanged (Figure 10, B and C). MIIG mice treated with CpG also showed less of an increase in a broad array of serum cytokines and chemokines implicated in cytokine storm, including TNF, IL-10, IL-18, IFN-γ, CXCL9, and CXCL10 (Figure 10D), while other cytokines such as IL-6 and GM-CSF showed similar upregulation. Together, we find that many systemic clinical features — including cytokine production in MAS, specifically — require macrophage responsiveness to IFN-γ, while others, including IL-6 induction and lymphopenia, were unaltered.

We then examined whether macrophage responsiveness to IFN-γ was required for pulmonary and airway inflammation. MIIG mice treated with CpG did not show any increase in IL-18, CXCL9, or CXCL10 in BAL fluid (Figure 10E). AMϕ isolated from MIIG mice treated with CpG to induce MAS and then cultured ex vivo failed to secrete CXCL9 after MAS induction (Figure 10F). Finally, AMϕ from MIIG mice showed reduced upregulation of several genes and microRNA associated with proinflammatory polarization, including *CXCL9*, *HMOX1*, *IL12A*, and miR-146a (Figure 10G). Taken together, experiments with IFN-γ blockade and MIIG mice demonstrate that macrophage responses to IFN-γ are required for pulmonary and airway inflammation in the CpG model of MAS, and they further support that systemic MAS induces pulmonary inflammation, reflecting key features of SJIA-LD in children.

## Discussion

The risk factors and underlying mechanisms driving often fatal SJIA-LD are unclear but have been proposed to include introduction of anticytokine therapies, allergic reactions, and recurrent MAS (15–17). This knowledge gap represents a key barrier to progress toward treating and preventing the condition. Here, our previously unreported findings of significant pulmonary and airway inflammation in a well-characterized mouse model of MAS contribute to improved understanding of SJIA-LD and potential therapeutic effects. Utilizing scRNA-seq, we found features of IFN pathway transcriptional activation throughout the lung during acute MAS, along with massive expansion of the myeloid compartment and emergence of MAS-specific macrophage populations, including recruited airway macrophages and a distinct IFN-γ–induced chemokine producing population. Histologic lung infiltrates are present acutely during MAS but become much more prominent after systemic MAS resolution. This is accompanied by large-scale changes in AMϕ activation and polarization from an M1 to an M2 phenotype. In contrast, with recurrent episodes of MAS, pulmonary inflammation in mice is exacerbated and associated with a reprogramming of AMϕ polarization from an antiinflammatory/resolution phenotype to a proinflammatory phenotype. Finally, pulmonary inflammation was ameliorated by direct IFN-γ blockade and requires macrophage responsiveness to IFN-γ. Together, our findings support CpG-induced MAS as a powerful model system for the study of SJIA-LD.

There are striking similarities between SJIA-LD and the findings presented herein (16). First, mice with MAS display chronic lymphocytic perivascular and peribronchiolar interstitial inflammation that is patchy and multifocal, resembling the interstitial component of SJIA-LD. Second, pulmonary inflammation in mice was more extensive after both systemic MAS resolution and during recurrent MAS, in agreement with patients developing signs of SJIA-LD in the months after disease onset. Third, both murine MAS and children with SJIA-LD display high levels of IL-18 in both serum and BAL. Fourth, BAL fluid in mice with MAS similarly displayed high levels of IFN-induced chemokines, which was also seen in some children with SJIA-LD. Finally and most strikingly, both pulmonary gene expression in murine MAS and in SJIA-LD reflected IFN-γ activation, a key pathogenic mediator of MAS. These findings support a shared pathogenic link between MAS and SJIA-LD.

Our gene expression profiling demonstrates large-scale reprogramming of AMϕ polarization phenotypes both during acute MAS and resolution. AMϕ in acute MAS activated gene pathways consistent with proinflammatory M1 polarization, including response to IFN-γ. In contrast, resolution phase AMϕ displayed more extensive transcriptional changes reflecting alternative activation and proresolution phenotypes, including increased *TGFβ* mRNA, STAT6 activation, and decreased expression of STAT1 targets (associated with IFN-γ) (33, 34). Interestingly, recurrent MAS rapidly reprogrammed AMϕ back to a proinflammatory polarization phenotype. Such alterations in AMϕ polarization are notable, given the association of SIJA-LD with PAP, caused by defects in AMϕ homeostatic functions and polarization (21, 35, 36). Systemic MAS resolution phase AMϕ also downregulated genes regulated by Bach1, a transcriptional repressor with key roles in limiting M2 phenotypes (37). Bach1 deficiency has been shown to worsen some forms of PAP, raising the intriguing possibility that failure to properly activate proresolution programs such as Bach1 during chronic/recurrent MAS could contribute to PAP (20).

Our findings also support a central role for IFN-γ in a broad array of inflammatory features in the lungs during MAS. There is increasing evidence in both animal models and patients for a key role of IFN-γ broadly in MAS pathogenesis (3) including the TLR9 mouse model (11, 13, 14). Our findings in MIIG mice extend these observations by showing that, specifically, macrophage responses to IFN-γ are required for full MAS features, including more pronounced reductions in some serum cytokines compared with those seen with anti–IFN-γ monoclonal antibody treatment. We also demonstrate broad transcriptional responses to IFN throughout the lungs, including emergence of MAS-specific macrophage populations with phenotypes suggesting tissue hemophagocytes, including apoptotic cell removal and response to IFN-γ. Notably, these phenotypes are similar to those we have recently reported in the bone marrow of a patient with early MAS (38). The present study also complements our recent findings in children with SJIA-LD and suggests that IFN-γ may also have a key role in lung disease pathogenesis (16). An anti–IFN-γ monoclonal antibody has been recently approved for familial HLH and is currently being investigated for treatment of MAS (NCT03311854, ClinicalTrials.gov). It remains to be seen whether such IFN-targeted therapy may be beneficial in SJIA-LD.

Our findings demonstrate chronic lymphocytic interstitial inflammation and IFN-driven transcriptional response in the lungs of mice with experimental MAS. However, despite signs of AMϕ activation, we did not identify features of PAP, which is a key pathologic feature of SJIA-LD (16). This suggests that MAS alone is not sufficient to cause SJIA-LD. Our findings would support a model where the cytokine milieu of MAS — including highly elevated IL-18 and IFN-γ — is a necessary but not sufficient risk factor for SJIA-LD development. Many SJIA-LD patients experience recurrent MAS or chronic, subclinical MAS. Given our above findings regarding reprogramming of AMϕ with recurrent MAS, the question arises as to whether SJIA-LD patients fail to activate resolution AMϕ phenotypes (including Bach1) that affect surfactant metabolism. Additionally, a crucial unknown regarding SJIA-LD is the possible role of cytokine-blocking biologic medications (anti–IL-1 and anti–IL-6), the introduction of which is temporally associated with emergence of SJIA-LD (17). IL-1 blockade can potentiate IFN responses in children with SJIA (39, 40); however, the effects of biologics on pulmonary inflammation remain unknown. Therefore, further work is urgently needed to define the pathogenic mechanisms connecting SJIA, MAS, and chronic lung disease.

## Methods

### Murine MAS induction.

Female C57BL/6J mice 6 weeks of age were purchased from The Jackson Laboratory. MIIG mice were obtained from Cincinnati Children’s Hospital Medical Center (32). Mice were treated with 5 i.p. doses of vehicle (PBS) or 50 μg CpG 1826 as described (Supplemental Figure 1) (11). For IFN-γ blockade, mice were given i.p. injections of isotype control or XMG1.2 in conjunction with CpG as described (11). Further details on mice, sample collection, histology, and immunohistochemistry are in the Supplemental Methods.

### Flow cytometry, cytokine determination, quantitative PCR, and microarrays.

Full experimental procedures are in the Supplemental Methods.

### scRNA-seq.

scRNA-seq experimental protocol and analysis are described in the Supplemental Methods.

### Statistics.

Data are presented as the mean ± SEM. PBS versus CpG comparisons were performed with 2-tailed Student’s *t* test; comparison between multiple groups were compared using 1-way ANOVA with follow-up Dunnett’s multiple-comparison test. For all tests, a *P* value less than 0.05 was considered significant.

### Study approval.

All studies were approved by the Cincinnati Children’s Hospital Medical Center IACUC.

## Author contributions

GSS designed the study. DKG, MH, ST, and GSS performed experiments. DKG, NS, CW, KT, AAG, ST, MBJ, KAWB, and GSS analyzed the data. GSS and NS wrote the first draft of the manuscript. All authors contributed to, reviewed, and approved the final version of the manuscript.

## Supplementary Material

Supplemental data

Supplemental tables 1-6

## Version 1 07/27/2021

In-Press Preview

## Version 2 09/08/2021

Electronic publication
