# Supplementary material for: IFN-γ is essential for alveolar macrophage–driven pulmonary inflammation in macrophage activation syndrome
Source: JCI Insight. 2021 Sep 8;6(17):e147593. doi: 10.1172/jci.insight.147593 (PMC8492332; doi:10.1172/jci.insight.147593)
Supplement: Supplemental data [file jciinsight-6-147593-s265.pdf]

IFNγ is essential for alveolar macrophage driven lung inflammation in macrophage activation syndrome

Data Supplement

Denny Gao[1], Nathan Salomonis[2,3], Maggie Henderlight[1], Christopher Woods[4], Kairavee Thakkar[3], Alexei A. Grom[1,2], Sherry Thornton[1,2], Michael B. Jordan[2,5], Kathryn A. Wikenheiser-Brokamp[4,6], Grant S. Schulert[1,2]

Divisions of [1]Rheumatology, [3]Biomedical Informatics, [4]Pathology & Laboratory Medicine, and [5]Immunobiology, Cincinnati Children's Hospital Medical Center and Departments of [2]Pediatrics and [6]Pathology & Laboratory Medicine, University of Cincinnati College of Medicine, Cincinnati, OH, USA

Supplemental Methods:

*Murine MAS induction***.** All studies were approved by the CCHMC Institutional Animal Care and Use Committee (IACUC). Female C57BL/6J mice 6 weeks of age were purchased from the Jackson Laboratory (Bar Harbor, ME). Macrophage insensitive to IFNγ (MIIG) mice were obtained from Dr. Michael Jordan at CCHMC (1). The TLR9 ligand CpG 1826 (Sequence: 5' TCCATGACGTTCCTGACGTT 3') was synthesized and purchased from InvivoGen (San Diego, CA), and the XMG1.2 monoclonal anti-mouse IFNγ from Bio X Cell (West Lebanon, NH). Mice were treated with five intraperitoneal doses of vehicle (Dulbecco's Phosphate Buffered Saline, DPBS) or 50 µg CpG 1826 on days 0, 2, 4, 7, and 9 (2). Mouse weight was recorded after each injection. Mice were sacrificed within twenty-four hours after the last injection (acute MAS) or allowed to recover for three weeks before either sacrifice (MAS resolution) or retreatment followed by sacrifice (recurrent MAS) (Figure S1). For IFNγ blockade, mice given IP injections of rat IgG1 isotype control antibody (Bio X Cell) or XMG1.2 in conjunction with CpG at a dose of 0.5 mg XMG1.2 on days 0 and 2, followed by 0.15 mg isotype control or XMG1.2 on days 4, 7, and 9, as described (2).

Sacrifice and Sample Collection. Mice were anesthetized with isoflurane and blood collected through cardiac puncture. Blood allowed to clot for 1hr on ice followed by centrifugation to collect serum. Where indicated, the right lung was removed, homogenized in 10ml PBS, sediment removed by centrifugation, and saved for cytokine determination. Spleens were collected to determine splenomegaly and bronchoalveolar lavage (BAL) fluid was collected through lavage of the lungs with 3 mL of 0.1 mM EDTA as described (3). Spleen and liver samples were collected from mice and immersion fixed in 10% formalin. Mouse lungs were inflated, removed, and immersion fixed in 10% formalin. All samples were processed, embedded, and stained by the CCHMC Pathology Research Core with hematoxylin and eosin (H&E) or immunohistiochemistry with antibodies against CD3 (2GV6, Roche,

Basel, Switzerland), CD68 (ab125212, Abcam, Cambridge, MA), B220 (ab10558, Abcam), or Pax5 (clone EPR3730, Abcam).

Lung cell dissociation. 25 mg fresh lung tissue was minced on ice and then shaken and triturated on ice for 9 minutes with 1.5 mL enzyme mix (90 μL of 100 mg/mL Collagenase A (Sigma), 150 μL of 43 U/mL lyophilized Elastase (Worthington), 150 μL of 90 U/mL purified neutral Protease (Worthington), 7.5 μL of 1M $CaCl_2$, 7.5 μL of 125 U/mL DNase I (Applichem), and 1095 μL of DPBS). After allowing to settle, the upper layer was filtered through a 30 μM filter and collected, followed by rinsing of filter with 0.04% BSA. Lich enzyme mix (1 uL of 0.5M EDTA, 100 μL of 100 mg/mL Bacillus Licheniformis (Sigma), and 899 μL DPBS) was added to remaining tissue and triturated on ice for 10 minutes before being filtered and collected. Filter was again rinsed with 0.04% BSA, after which samples were lysed with RBC Lysis solution before being resuspended in 0.04% BSA.

Immunohistochemistry quantitation. Immunohistochemically stained, glass histology slides were scanned at 40x using a Leica Aperio AT2 instrument. The Aperio nuclear staining algorithm was run on test digitalized slides and the algorithm parameters modified to accurately score positively stained cells. The algorithm was then run on digitalized images of anti-CD3 immunohistochemically stained lung sections from mice treated with PBS or CpG as above. Lung histologic sections were demarcated on the slides for calculation of tissue area. Cell staining was classified based upon staining intensity with positive cells scored as red (3+), orange (2+) or yellow (1+) and negative cells scored as blue (0). Data are represented as positive cells (combined1+ to 3+ or 2+ and 3+ only) per mm^2 of lung tissue.

BAL Processing. After collection, BAL cells were counted, centrifuged, and cell-free BAL fluid stored at -80°C. BAL cells were resuspended in RMPI, and AMϕ were purified by adherence for 1hr followed by sequential washing to remove non-adherent cells. AMϕ were either fixed for use in flow cytometry, RNA extracted with Trizol, or incubated in fresh media at 37°C for chemokine release.

Flow Cytometry. Cells were incubated with Brilliant Stain Buffer and antibodies for thirty minutes. All antibodies were obtained from BD Biosciences except anti-CD206 which was from BioLegend. BAL cells were stained with FITC-conjugated anti-CD11b (clone M1/70), PE-Cy7-conjugated CD11c (clone HL3), PE-conjugated anti-CD64 (clone X54-5), BV786-conjugated anti-I-A/I-E (MHC class II; clone M5/114), AF700-conjugated CD24 (clone M1/69), BV711-conjugated Ly6C (clone 1A8), and AF647-conjugated anti-CD206 (clone C068C2). Blood monocytes were stained with BV711-conjugated anti-Ly6G and PE-conjugated anti-CD64. Samples were analyzed using LSR Fortessa and analyzed using BD FACSDiva. Sequential gating strategy (Figure S10) was used to isolate target cell populations (4) including $CD11c^{+}CD11b^{var}CD64^{+}$ and $CD11c^{-}CD11b^{var}$ macrophages (5).

Blood Monocyte Processing. Blood was collected into tubes with K_2EDTA and the hematology profile determined using Hemavet 950 FS (Drew Scientific Inc.). Blood was lysed with RBC Lysis solution. Cells were resuspended in RPMI Medium and incubated at 37°C to allow monocytes to adhere. After 1hr, cells were washed to remove non-adherent cells and RNA was then extracted via Trizol.

Cytokine determination. Cytokine concentrations were determined by using Milliplex™ Multiplex kits (MilliporeSigma, Darmstadt, Germany) as described (6). Levels of IL-18 (MBL International Corporation,

Nagoya, Japan), ferritin (ALPCO, Salem, NH), CXCL9, and CXCL10 (R&D Systems, Minneapolis, MN) were determined by ELISA per provided protocols.

Quantitative PCR. RNA samples were analyzed as previously described (7) and normalized to GAPDH for coding genes or sno135 for microRNA. Data was collected and analyzed using the QuantStudio 3 Real-Time PCR System (Applied Biosystems). Primer sequences are in Table S11.

Single-cell RNA sequencing Following dissociation, cells were diluted to 1,000 cells/L, and 15,000 cells from wild-type and MAS mice were each loaded in a separate lane of a Chromium Controller Instrument (10x Genomics). The 10x Genomics single cell 3' v3 assay protocol was followed as described until the cDNA amplification step. Sequencing was performed on the Illumina NovaSeq (Novogene). Single-cell RNA-Seq analyses were performed as recently described (8). In brief, the 10x Genomics libraries were aligned to the mouse genome (mm10), with unique molecular index quantified (UMI) gen counts obtained using the Cell Ranger workflow (version 3.1.0) using the mm10-2.1.0 reference transcriptome. Each sample was sequenced to a depth of >390 million reads, with over 9,600 called cell barcodes from each library, with >34,000 UMI per cell on average (see summary metrics in Table S1). Using the Cell Ranger filtered cellular barcodes, the associated sparse-filtered HDF5 files were jointly processed (WT and MAS) in AltAnalyze and ICGS2 (Iterative Clustering and Guide-gene selection version 2) to obtain normalized gene counts (counts per ten thousands (CPTT) UMIs) and initial predicted cell populations (9). ICGS2 was run using the default options (cosine clustering) in addition to a PageRank down-sampling threshold of 5,000 cells. ICGS2 identified two cell populations (c20 and c34) as presumptive unknown cell types (no enrichment in its BioMarkers gene set database), that were excluded prior to downstream analyses due to a lack of protein-coding marker genes (only lncRNAs) and broad UMAP dispersion. Cell populations

names were revised from the original ICGS2 predictions based on the literature (marker gene driven). The top-specific marker genes for each Macrophage and Monocyte cluster were derived using the MarkerFinder algorithm and gene set enrichments using GO-Elite, both AltAnalyze (9). Comprehensive differential expression and gene regulatory prediction analyses were performed using these alignment results in the software cellHarmony with the supplied ICGS2 groups for both WT and MAS (options --referenceType None) and eBayes $p<0.05$ FDR adjusted and fold>1.2 (10). Included UMAP plots were produced by cellHarmony or downstream by AltAnalyze by querying the expression of individual genes (--genes "<gene symbols>" --separateGenePlots True). The single-cell genomics data has been deposited in GEO (GSE169325 - reviewer access token ojslcqwclbylpkd).

Microarray. Whole transcriptome analysis of AM[illegible] was determined using microarray. Briefly, RNA was extracted from purified AMϕ as described above. A minimum of 500 picograms of RNA was utilized to construct library using the Clariom S Pico Kit (Affymetrix), after which samples were run on the Clariom S Array (Affymetrix). Arrays were imaged using GeneChip Scanner 3000 7G (Affymetrix), and relative signal value and linear value were calculated for all probe sets. Data was subsequently analyzed using AltAnalyze to determine differentially expressed genes and perform pathway analysis. Microarray data has been deposited in Gene Expression Omnibus (GSE149034).

Supplemental References:

1. Lykens JE et al. Mice with a Selective Impairment of IFN-γ Signaling in Macrophage Lineage Cells Demonstrate the Critical Role of IFN-γ–Activated Macrophages for the Control of Protozoan Parasitic Infections In Vivo [Internet]. *J. Immunol.* 2010;184(2):877–885.

2. Behrens EM et al. Repeated TLR9 stimulation results in macrophage activation syndrome-like disease in mice [Internet]. *J Clin Invest* 2011;121(6):2264–2277.

3. Van Hoecke L, Job ER, Saelens X, Roose K. Bronchoalveolar Lavage of Murine Lungs to Analyze Inflammatory Cell Infiltration [Internet]. *J. Vis. Exp.* [published online ahead of print: May 4, 2017];(123). doi:10.3791/55398

4. Misharin A V, Morales-Nebreda L, Mutlu GM, Budinger GRS, Perlman H. Flow cytometric analysis of macrophages and dendritic cell subsets in the mouse lung. [Internet]. *Am. J. Respir. Cell Mol. Biol.* 2013;49(4):503–10.

5. Redente EF et al. Tumor necrosis factor-α accelerates the resolution of established pulmonary fibrosis in mice by targeting profibrotic lung macrophages. [Internet]. *Am. J. Respir. Cell Mol. Biol.* 2014;50(4):825–37.

6. Hinze CH et al. Immature cell populations and an erythropoiesis gene-expression signature in systemic juvenile idiopathic arthritis: implications for pathogenesis. [Internet]. *Arthritis Res. Ther.* 2010;12(3):R123.

7. Schulert GS et al. Monocyte MicroRNA Expression in Active Systemic Juvenile Idiopathic Arthritis Implicates MicroRNA-125a-5p in Polarized Monocyte Phenotypes. *Arthritis Rheumatol.* 2016;68(9). doi:10.1002/art.39694

8. Muench DE et al. Mouse models of neutropenia reveal progenitor-stage-specific defects. [Internet]. *Nature* 2020;582(7810):109–114.

9. Venkatasubramanian M, Chetal K, Schnell DJ, Atluri G, Salomonis N. Resolving single-cell heterogeneity from hundreds of thousands of cells through sequential hybrid clustering and NMF. [Internet]. *Bioinformatics* 2020;36(12):3773–3780.

10. DePasquale EAK et al. cellHarmony: cell-level matching and holistic comparison of single-cell transcriptomes [Internet]. *Nucleic Acids Res.* 2019;47(21):e138–e138.

Supplemental Figures

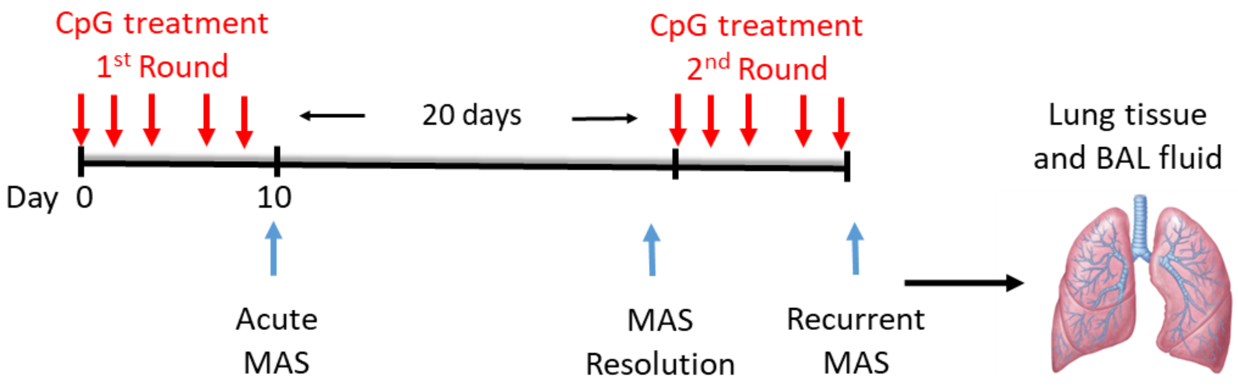

Supplemental Figure 1: Experimental overview. C57Bl/6 mice are given intraperitoneal injections of CpG on days 0, 2, 4, 7, and 9. They are then either sacrificed on day 10 (acute MAS time point) or allowed to recover until day 30. At that time they are either sacrificed (MAS resolution) or retreated as before with 5 CpG injections prior to sacrifice (recurrent MAS).

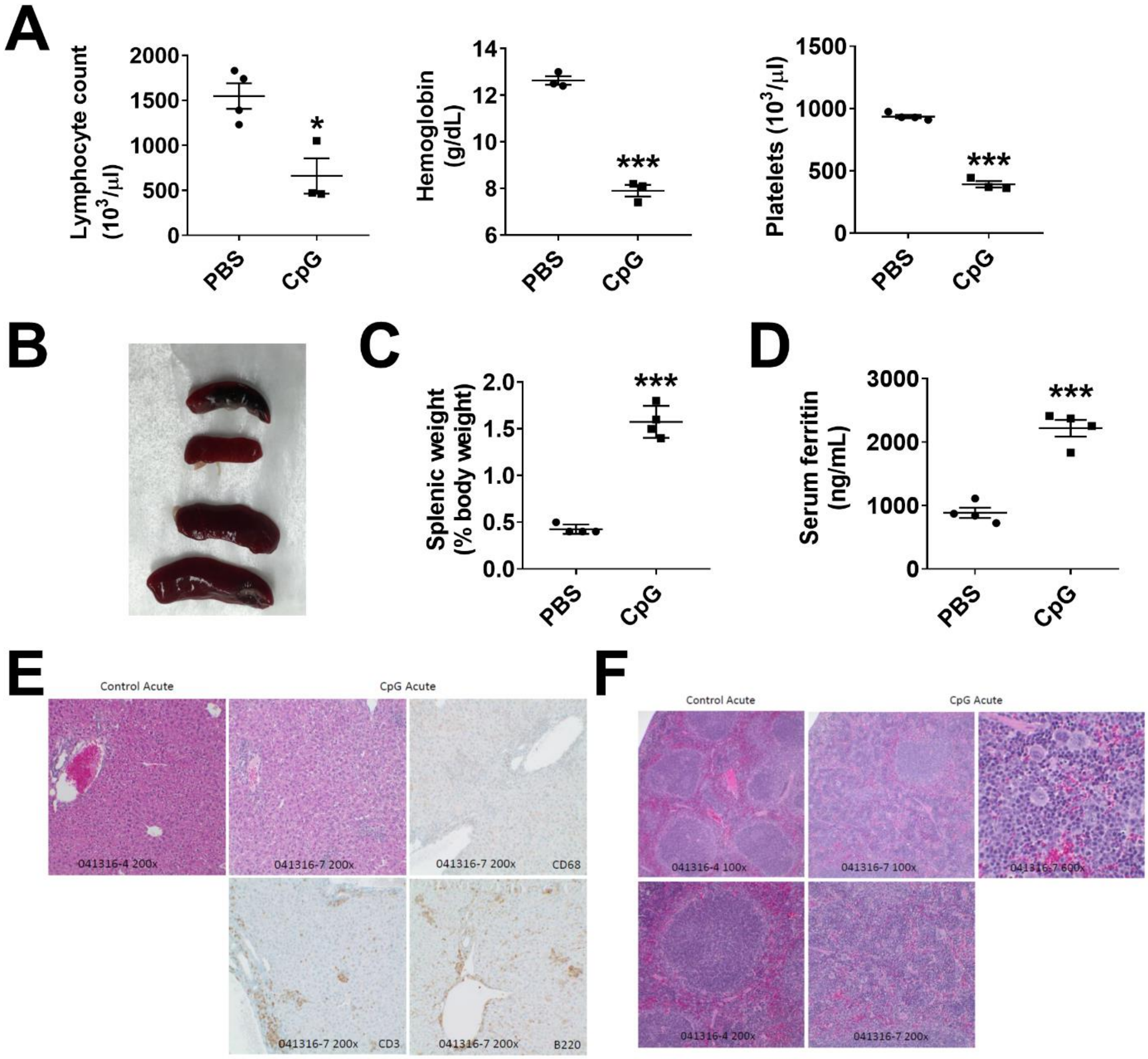

Supplemental Figure 2: CpG treatment induces clinical features of macrophage activation syndrome. A, CpG treated mice demonstrate mild lymphopenia (left), anemia (center), and thrombocytopenia (right) compared to mice treated with PBS alone. B-C, CpG treated mice have marked splenomegaly. D, CpG treatment induces serum hyperferritinemia. E-F, liver (E) and spleen (F) histology for control (PBS) or CpG treated mice during acute MAS. Sections stained with H&E or with immunostains for CD3, CD68, or B220 as indicated.

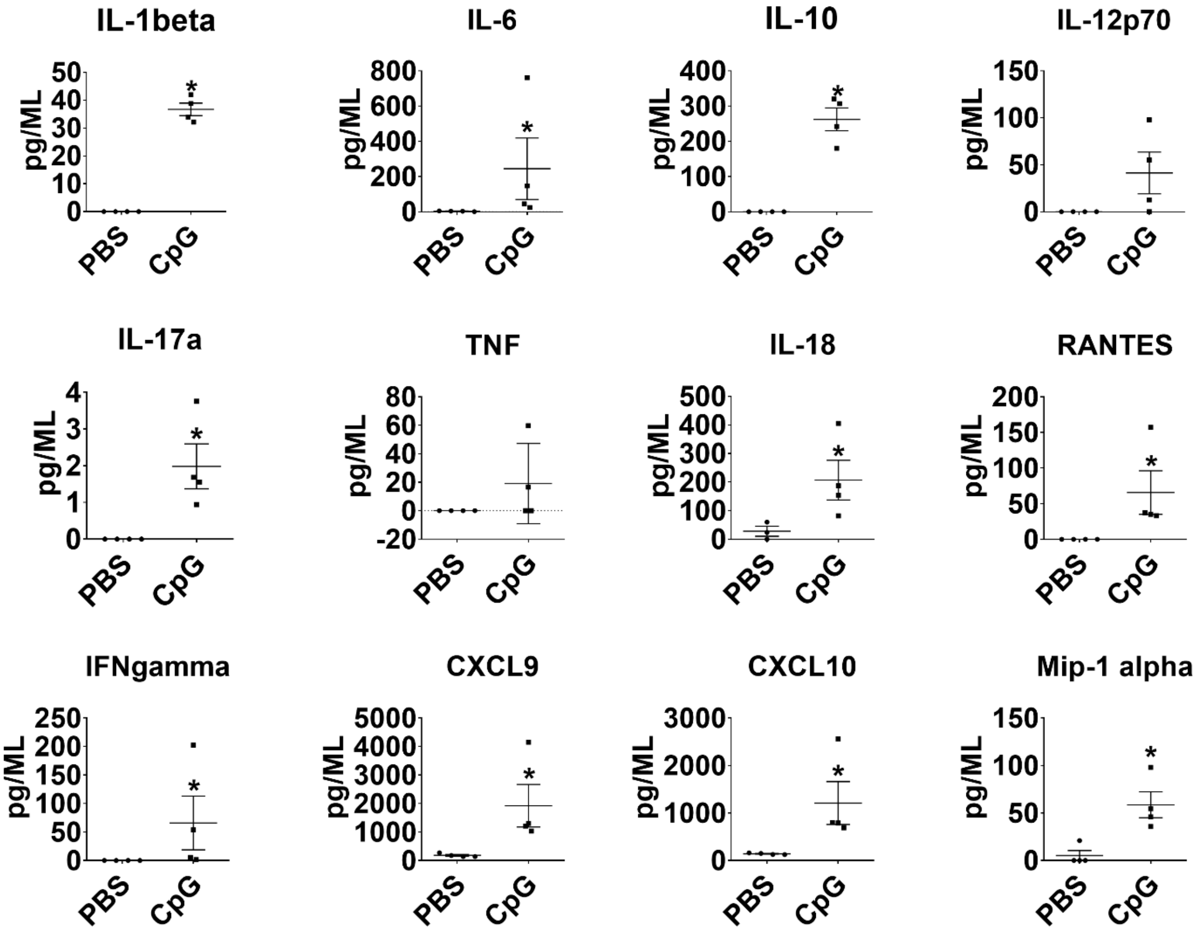

Supplemental Figure 3: CpG treated mice demonstrate marked elevations in serum cytokines and chemokines. Cytokine levels were determined by Milliplex Multiplex kits, except for IL-18 which was by ELISA. Data are representative of three independent experiments (n=4). Error bars represents mean±SEM. *p<0.05.

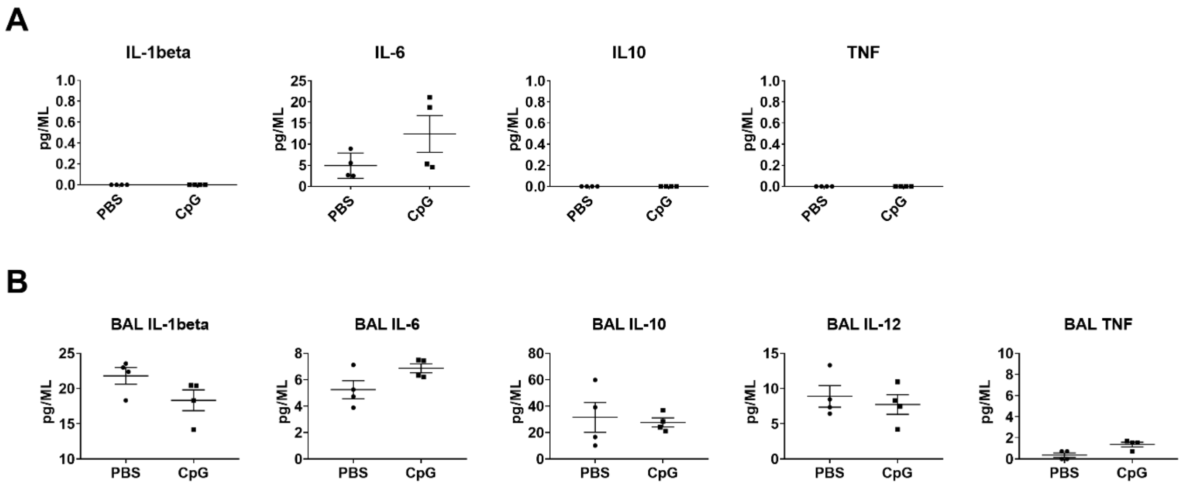

Supplemental Figure 4: Normal levels of proinflammatory cytokines in (A) whole lung digests and (B) BAL fluid from mice with acute MAS. Data are representative of three independent experiments (n=4). Error bars represents mean±SEM.

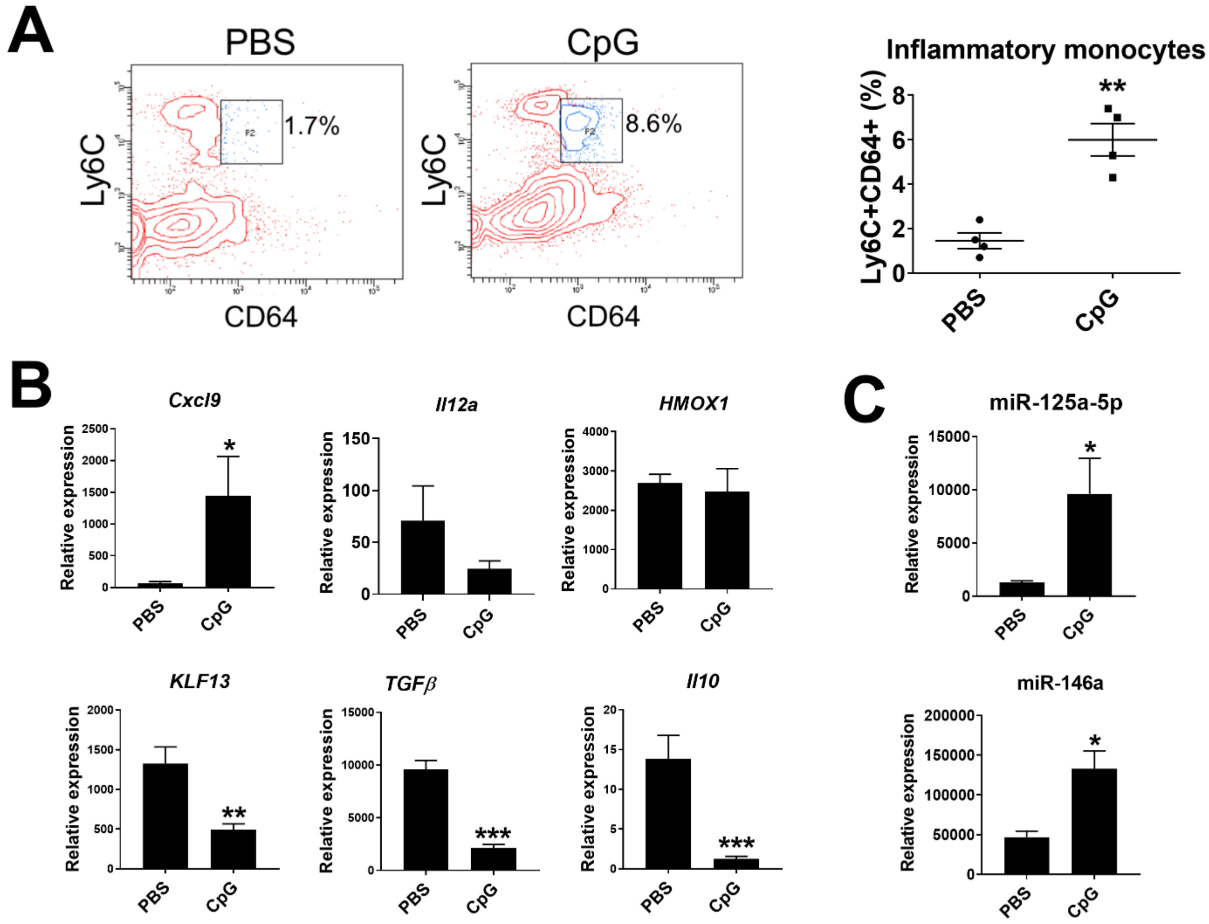

Supplemental Figure 5: Phenotype of blood monocytes during acute phase of MAS. A, Blood monocyte inflammatory monocyte population as determined by flow cytometry. B, Blood monocyte gene expression as determined by qRT-PCR. C, Blood monocyte microRNA expression. Data are representative of three independent experiments (n=4). Error bars represents mean±SEM. *p<0.05, **p<0.01, ***p<0.001.

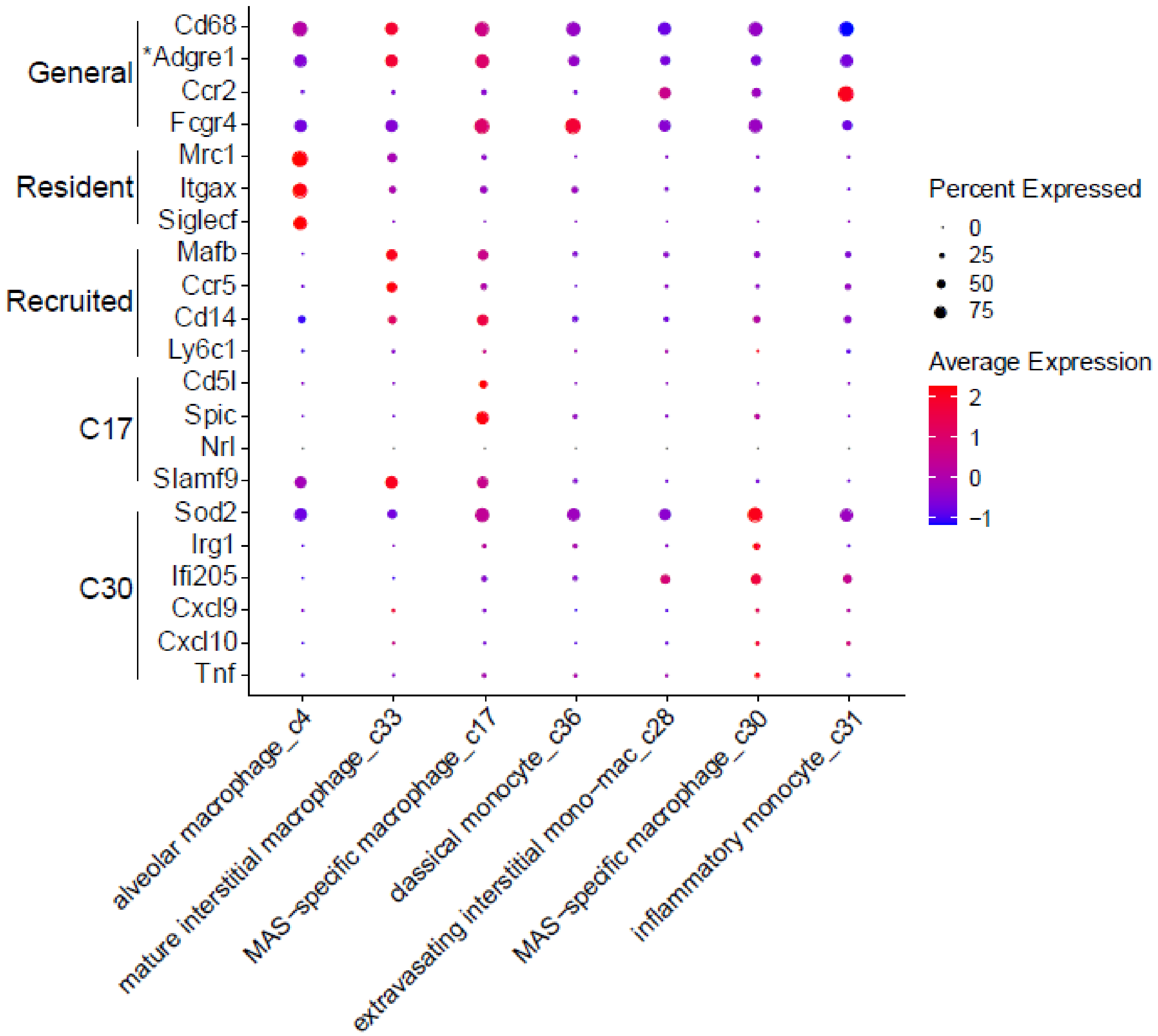

Supplemental Figure 6: Relative expression of macrophage marker genes in distinct monocyte/macrophage subsets in mouse lungs during MAS. Dot color shows relative average gene expression for all cells in the cluster compared to all other cells. Sizes of dot shows number of cells in the cluster expressing the gene.

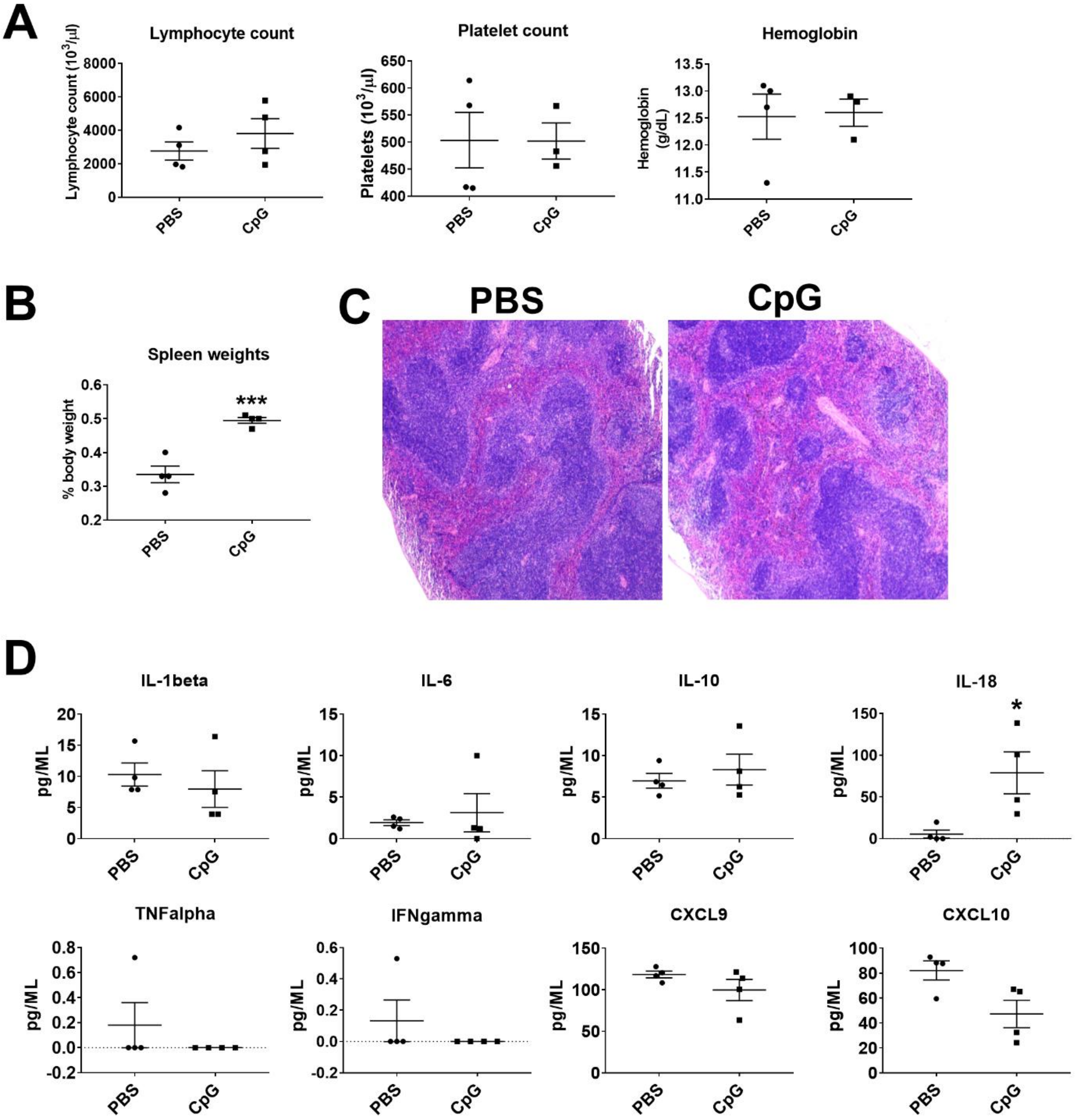

Supplemental Figure 7: Systemic features of recovery 3 weeks after MAS (MAS resolution). A, CpG treated mice no longer demonstrate lymphopenia, thrombocytopenia, or anemia compared to PBS treated mice after recovery. B-C, CpG treated mice still demonstrate mild splenomegaly, though significantly less than seen in acute MAS. Splenic architecture is largely restored but there is continued expansion of the red pulp. D, Serum cytokine profile. Data are representative of three independent experiments (n=4). Error bars represents mean±SEM. *p<0.05, **p<0.01, ***p<0.001.

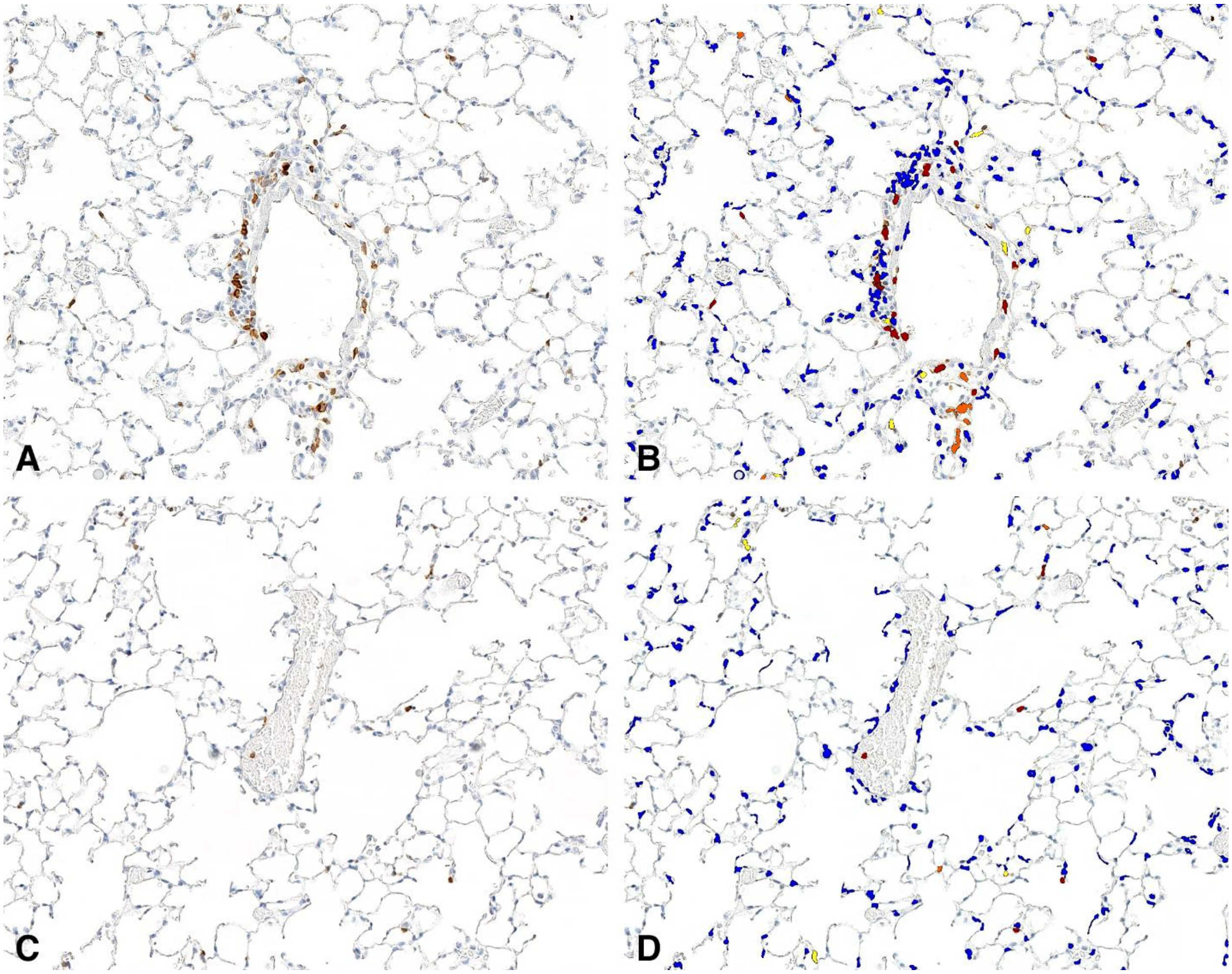

Supplemental Figure 8: Quantitation of CD3+ cells in lung tissue. A-B represents a treated mouse and C-D are images from a control animal. A and C represent anti-CD3 IHC stained sections. Cell staining was classified into categories based upon staining intensity with positive cells scored as red (3+), orange (2+) or yellow (1+) and negative cells scored as blue (0) (B and D). Data are represented as positive cells (combined 1+ to 3+) per mm^2 of lung tissue.

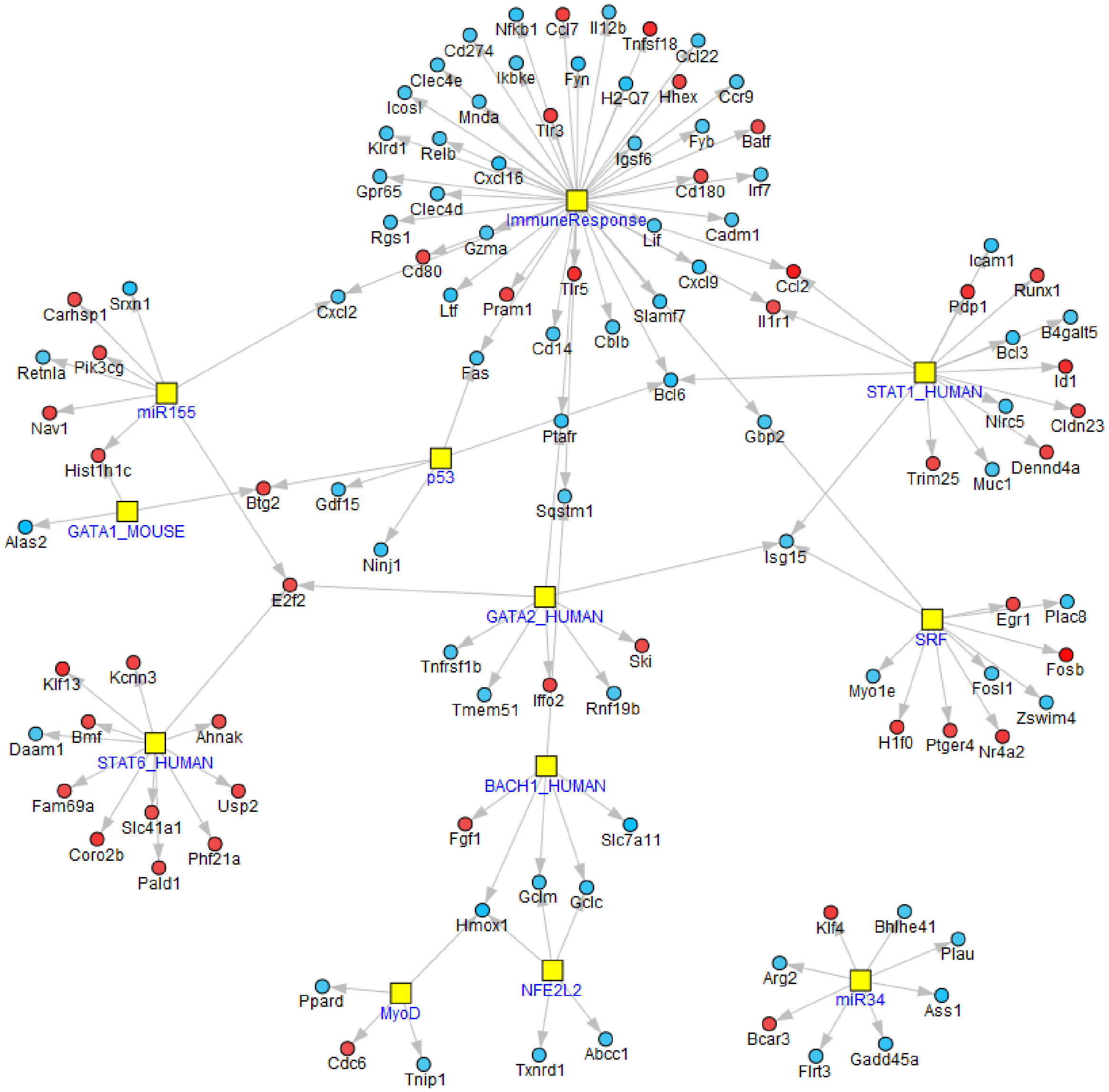

Supplemental Figure 9: Statistically enriched transcription factor predictions of differentially expressed genes in alveolar macrophages during MAS resolution vs. control. Transcription factor targets from the GO-Elite TFTarget database (combined PAZAR, TRRUST, Amadeus databases), based on ChIP evidence. Data visualization generated by GO_Elite in AltAnalyze. Red=upregulated, blue=downregulated.

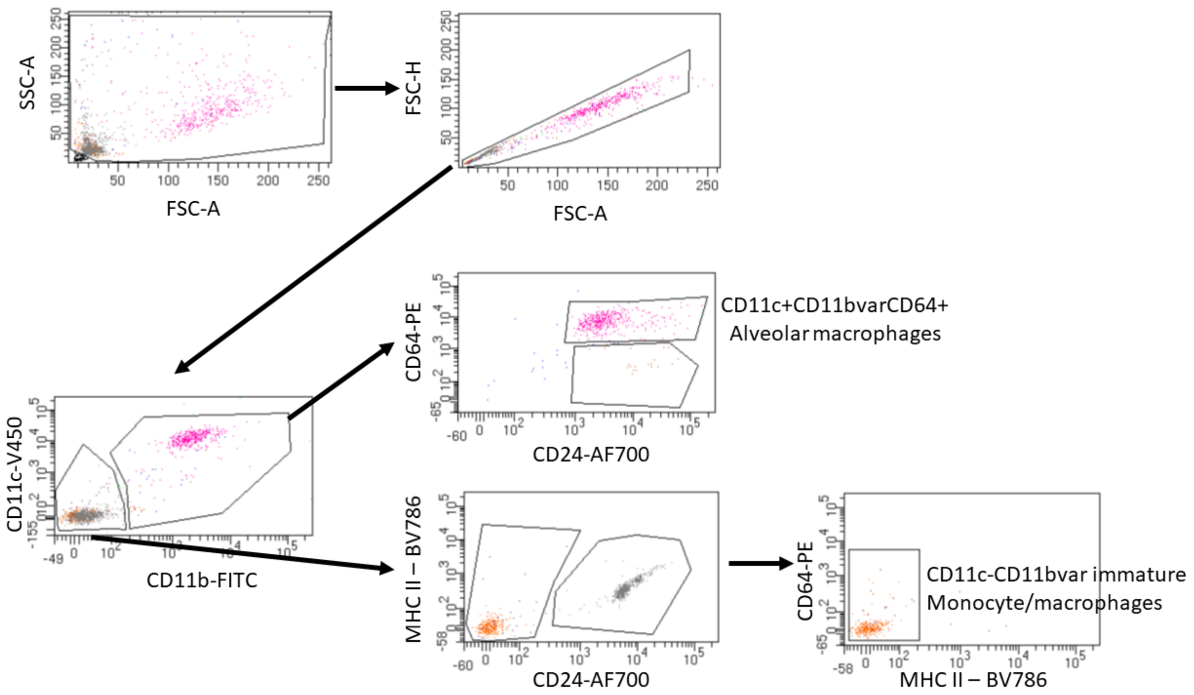

Supplemental Figure 10: Alveolar macrophage flow cytometry gating strategy. Cells were gated and doublets removed via FSC-A vs FSC-H. Cells were then gated for CD11c^{+}CD11b^{var} and CD11c^{-}CD11b^{var} populations. CD11c^{+}CD11b^{var} cells were then gated on CD64 vs CD24 to remove granulocytes (CD24^{+}) and yield CD11c^{+}CD11b^{var}CD64^{+} alveolar macrophages. CD11c^{-}CD11b^{var} cells were gated on CD24^{-} cells to identify immature monocytes/macrophages from CD24+ granulocytes.

Supplemental tables

Table S7: Differentially expressed genes upregulated in AMϕ during acute MAS in PBS (control) vs CpG (MAS) mice (p<0.05, fold change >2.0)

Gene	Average Control	Average MAS	Fold change (MAS_vs._Control)	P value (MAS_vs._Control)
Hba-a2	12.14128	17.10266	31.15477	0.001352
Hba-a1	12.2778	16.98512	26.12428	0.001175
Alas2	6.084054	10.46079	20.77443	0.015998
Hbb-bt	13.99633	17.66566	12.72267	0.002004
Hbb-bs	14.12558	17.74891	12.32344	0.002878
Bpifb1	6.784819	9.906384	8.703319	0.005455
Cxcl9	5.412461	8.133263	6.592395	3.71E-06
Tspan1	6.056071	8.745515	6.450649	1.36E-05
Scgb3a1	8.297481	10.94589	6.269738	0.000279
Bpifa1	9.807678	12.3575	5.855605	0.000305
Cyp2a5	7.141375	9.660456	5.732167	0.000723
Scgb3a2	10.80422	13.27295	5.535585	0.000157
Scgb1a1	8.92432	11.15798	4.703264	0.000168
Il2rb	4.959077	7.031341	4.20546	0.000219
H2-Q7	8.114265	10.17965	4.185464	2.51E-06
Gbp2	5.829648	7.813423	3.955268	4.19E-06
Ctla2a	6.990299	8.950457	3.891044	4.64E-05
Ly6c1	4.649626	6.574279	3.796455	6.07E-06
H2-Q5	9.299653	11.22332	3.793859	0.000217
Ube2l6	8.009255	9.914891	3.74674	0.024667
Cbr2	8.222673	10.12131	3.728612	1.81E-06
C1qa	7.723405	9.607021	3.689989	1.60E-05
8030474K03Rik	5.036066	6.773091	3.33347	0.014138
Fam26f	6.337433	8.016681	3.202608	7.19E-06
Nlrc5	5.113567	6.755964	3.12184	3.56E-05
Plac8	4.191997	5.756762	2.958293	0.016319
H2-Q8	8.889223	10.42974	2.90899	0.000905
Kit	7.011459	8.534283	2.87353	0.000662
Cyp2f2	7.441815	8.923321	2.792402	0.000258
Ifi203	7.86559	9.335067	2.769214	1.32E-05
AW112010	11.75621	13.19015	2.701833	5.99E-05
Psmb9	12.27348	13.69129	2.671801	1.51E-05
Aif1	5.243725	6.641754	2.635414	0.011234
Ptp4a3	6.33388	7.710011	2.595714	0.024683

Tmem176b	7.469413	8.812588	2.537091	6.75E-05
Mndal	10.74967	12.05608	2.473245	9.25E-06
Gbp10	5.13625	6.437071	2.463691	0.000784
Gbp3	6.110736	7.398963	2.442278	0.000659
Epb41	12.24106	13.52683	2.438123	0.046462
Ly6c2	4.339217	5.624369	2.437077	0.000957
Plbd1	8.412211	9.67778	2.40422	0.003802
Ltf	5.743214	6.994841	2.381098	0.002145
Muc1	6.434777	7.64567	2.31481	0.001541
Tmem56	4.215347	5.396113	2.266971	0.002237
H2-Q4	6.210775	7.38169	2.251545	2.46E-05
Pigr	4.854699	6.025213	2.250919	0.005682
Irf1	7.588671	8.708013	2.172478	0.000644
Gm8279	4.415887	5.522926	2.154031	0.003237
Gm10413	4.415887	5.522926	2.154031	0.003237
Gvin1	10.53484	11.62899	2.134864	0.000225
Tap1	11.51842	12.60928	2.130002	1.90E-05
Klrd1	6.409701	7.497624	2.125678	0.001942
Nod2	6.698669	7.776296	2.110563	0.003356
Zdhhc7	9.305109	10.37501	2.099282	0.040433
St3gal5	4.945244	6.015044	2.099143	0.003811
Il12b	6.78647	7.855902	2.098608	0.000892
Gbp7	8.565078	9.62739	2.088276	0.000458
Psmb8	12.8829	13.93571	2.074563	4.49E-05
Gpr65	7.075792	8.126175	2.071079	0.000446
Casc4	4.449315	5.484371	2.049193	0.000579
Lap3	9.119898	10.14968	2.041719	0.006383
Olfr73	4.222863	5.252144	2.041007	0.006088
Ifi47	8.666091	9.689007	2.032022	6.79E-05
Cxcl14	7.810523	8.824875	2.019996	0.001937

Table S8: Downregulated genes in alveolar macrophages in PBS (control) vs MAS (CpG) (p<0.05, fold change >2.0).

Gene	avg-Control	avg-CpG	fold-CpG_vs_Control	p-CpG_vs_Control
Il1f9	9.058848	6.686785	-5.17681	0.000857
Gm21860	10.65596	8.902203	-3.37235	0.003315
Gm21748	10.65596	8.902203	-3.37235	0.003315
Slc18b1	6.106505	4.588521	-2.86391	0.003162
Olfr115	6.383395	5.036046	-2.54444	1.44E-06
Slc43a3	8.293362	6.984747	-2.47704	0.014527
Gm21887	14.30579	13.02405	-2.43132	0.049007
Myo5a	9.374405	8.119084	-2.3872	0.007423
Me1	6.986844	5.748878	-2.35866	0.000338
Ddah1	7.277507	6.043011	-2.35299	0.025566
Ptgr1	10.44556	9.238632	-2.30845	0.001203
Slc11a1	11.09123	9.899586	-2.28413	0.007269
Colec12	10.3089	9.134507	-2.25698	0.000224
Lrp8	10.95637	9.785393	-2.25163	0.001875
Vmn1r31	5.751948	4.584323	-2.24642	0.000251
Pcdhgb1	10.0502	8.935093	-2.16612	0.000176
Galnt6	10.19657	9.082887	-2.16397	0.000205
Mamdc2	8.539443	7.442267	-2.13936	9.96E-06
Ppard	12.78765	11.69492	-2.13277	0.000335
Frmd4a	8.487607	7.412059	-2.10752	1.22E-05
Adamtsl5	7.66265	6.590461	-2.10262	0.046566
Utp14b	8.032787	6.966982	-2.09334	0.031281
B430306N03Rik	9.10388	8.042923	-2.08632	0.039945
Ptgir	10.43227	9.390494	-2.05876	0.009414
Rnf128	9.307956	8.266285	-2.05861	0.000653
Tubb2a	12.27098	11.2323	-2.05435	0.000438
Klk1b11	7.224917	6.191764	-2.04649	0.000629
Slc40a1	9.163085	8.140691	-2.03129	0.006
Akr1b8	12.3622	11.35753	-2.00648	0.003884
Chpf2	11.58073	10.58011	-2.00085	0.00196

Table S9: Differentially expressed genes in alveolar macrophages during MAS resolution vs acute MAS (CpG) (p<0.05, fold change >2.0)

Gene	avg-Control	avg-Resolution	fold-Resolution_vs_CpG	p-Resolution_vs_CpG
Gm10800	8.395242	11.99956	19.02791	0.008109
Fosb	9.13404	13.41603	13.9973	2.65E-07
Gm10720	6.019691	8.986745	12.18037	0.003043
Sgms1	8.681099	11.0268	9.540038	7.26E-08
Ccl2	6.343016	9.313512	8.058326	1.59E-07
Gm10801	7.727279	10.59462	7.830205	0.017857
Gm11168	7.34275	9.911827	7.72477	0.021847
Gm10715	5.589161	8.081011	7.452188	0.020273
Tifab	8.962414	12.06282	7.364254	1.66E-07
Gm17535	6.579434	9.121814	7.121965	0.0325
Ffar2	6.125287	9.090715	6.422385	2.44E-08
Themis2	8.452516	11.74425	5.848402	1.22E-07
Ndst1	8.66308	10.67659	5.565398	2.99E-08
Trerf1	7.204388	9.776145	5.456901	2.21E-07
Trim47	5.717745	8.3268	5.434181	4.79E-06
Gapt	5.669644	7.84511	5.427612	1.05E-08
Abcd2	10.95687	13.11744	5.403529	7.99E-09
C5ar2	7.545385	10.1197	5.314219	3.91E-08
Tsc22d3	8.122904	10.28627	5.279877	5.82E-07
Il6ra	10.93396	13.88692	5.253334	5.50E-07
Rcan1	11.61312	14.35217	5.237266	2.81E-08
Id1	10.03239	12.74833	5.196226	2.07E-07
Pold1	8.639458	10.98253	5.189581	6.21E-06
Fzd4	7.509589	9.567807	4.992387	6.93E-07
Slco2b1	10.10302	12.02764	4.906879	1.46E-08
Stk38l	8.55415	10.92347	4.842142	1.88E-07
Tlr5	8.612325	10.60461	4.754056	3.41E-09
Tnfsf18	5.837503	7.598432	4.700852	0.001058
Gm10721	7.690289	9.947061	4.565513	0.029484
Pdgfc	10.18584	11.92147	4.511886	7.50E-08
Pdp1	7.565951	9.161541	4.482702	8.49E-07
Cbx4	8.667838	11.24661	4.420431	1.58E-05
Per2	6.023165	8.64585	4.38867	1.64E-06
P2ry6	8.271795	10.51206	4.372237	4.98E-07
Gpr160	8.025686	10.12336	4.365117	4.26E-07
Klf13	11.26526	13.63511	4.334091	1.87E-07
Tacc2	7.549098	9.498794	4.171922	3.42E-08
Krt19	12.01023	13.78905	4.023693	8.79E-08

Coro2b	8.646309	10.55078	3.969789	2.63E-07
Gm10718	7.155674	8.935595	3.96724	0.024529
5430427O19Rik	7.598758	9.514924	3.806246	5.72E-07
Midn	10.59729	12.62143	3.752946	9.64E-07
Serpine1	11.7662	13.61618	3.730457	3.64E-08
Slc16a6	10.52423	11.99584	3.698844	1.92E-05
AI504432	12.24038	14.28329	3.610874	1.61E-07
Synpo	6.281356	8.353794	3.573999	3.27E-08
4930503L19Rik	8.147876	9.966942	3.527667	3.86E-05
Nr4a2	8.756976	11.26829	3.505522	9.11E-05
Gm6377	9.408805	11.65742	3.496845	3.05E-08
Txnip	13.56992	15.53087	3.484742	0.000122
Fam89a	6.247726	8.07928	3.461642	0.000371
Cnksr3	8.057114	9.583654	3.437097	5.80E-06
Rgs2	11.00926	13.24761	3.426441	3.70E-05
Klf4	8.923638	10.95743	3.411069	2.95E-07
Erf	8.199091	10.03167	3.365515	6.83E-06
Zfp36l2	6.784689	8.78166	3.277559	7.71E-07
Kcnq1ot1	8.729242	9.831704	3.225899	0.000851
Dusp7	7.856492	9.93652	3.224289	3.17E-05
Arhgap32	6.150835	7.497315	3.201367	0.0002
Slc18b1	6.106505	6.262085	3.190016	0.000257
Zbtb34	7.823238	8.989897	3.170072	6.14E-05
Tet2	11.13729	12.96389	3.167816	9.92E-06
Stard9	6.515368	8.340863	3.164317	1.04E-05
Adgrg1	8.31749	9.617467	3.163732	5.70E-05
Pced1b	6.413896	7.715095	3.137298	5.79E-06
Pisd-ps3	10.12617	10.92739	3.114882	0.00022
A730008H23Rik	10.72191	12.02665	3.100034	2.02E-06
Tmem216	8.989765	10.55924	3.0744	2.23E-05
Gm1966	9.628675	11.70489	3.062415	2.01E-06
H1f0	7.462574	8.942643	3.042487	0.000178
Dgkg	7.735572	9.07554	3.038888	2.06E-05
Arl13b	8.030826	9.704408	3.03871	2.54E-05
Tcf19	6.053377	7.122502	3.030051	0.000301
Zfp398	7.071173	8.524949	3.027483	1.05E-05
Gabbr1	7.706388	8.943098	3.021002	4.94E-06
Ffar4	9.964034	11.52557	3.007886	3.28E-07
Amd2	8.716283	9.645373	2.980908	2.29E-05
Ccl7	5.609676	6.98692	2.980131	1.99E-06
Aloxe3	9.224097	9.99436	2.971987	0.000117

Tlr3	8.9147	10.62402	2.921369	2.02E-05
Slc9a3r2	6.441323	8.304478	2.905515	0.00029
Nr4a1	11.94384	14.03652	2.899182	6.79E-05
Slc39a10	9.753528	11.22083	2.896667	4.61E-05
Kcna3	9.148038	10.70752	2.895945	1.98E-06
Zfp608	10.00916	11.69019	2.871744	5.16E-07
Atp10d	5.851009	7.34223	2.86501	8.09E-06
Abcg1	12.77786	14.19789	2.842613	2.00E-06
Btg2	8.937113	10.44262	2.82725	0.00042
Zfp512b	9.572843	10.76102	2.817964	2.11E-05
Slc6a4	9.12436	10.07338	2.815169	1.86E-06
4930562F07Rik	8.930194	10.3164	2.799372	7.56E-06
Pram1	7.132391	8.27184	2.785198	2.27E-06
Agfg2	9.314099	11.04024	2.760305	3.67E-06
Batf3	6.35276	7.942701	2.741853	0.000176
Cped1	8.724445	10.40599	2.732322	7.82E-05
Zyx	8.800742	10.3133	2.722552	3.81E-07
Cldn23	8.086357	8.723986	2.705619	2.21E-05
Nav1	8.164392	9.453012	2.693399	7.78E-05
Gm9821	7.709958	8.599627	2.691271	0.000768
Gm21897	10.67344	11.46159	2.690707	0.00213
Hhex	5.277676	6.554465	2.687479	2.36E-05
Klf10	9.121991	10.409	2.679431	1.67E-06
Car11	6.27017	7.21555	2.673675	0.001035
Jarid2	9.209299	11.16542	2.669825	2.99E-06
Kbtbd7	9.666576	11.31289	2.669432	1.16E-05
Gm21962	10.88854	11.61061	2.665038	0.002289
Angptl4	7.115369	9.184384	2.662681	0.000186
AI467606	7.872421	9.156961	2.658045	2.06E-06
Egr1	9.289714	10.7545	2.636882	0.00012
Rassf5	12.38572	14.13041	2.632907	7.65E-06
Mamdc2	8.539443	8.838054	2.631322	1.14E-05
Vsig10l	7.885415	9.148718	2.629437	4.90E-07
Fam189a2	10.09496	11.05853	2.623611	6.30E-05
Mak	10.13349	11.23042	2.618422	7.60E-07
Tgm1	6.151825	7.857199	2.59685	1.69E-05
Kcne3	9.278967	11.16256	2.584636	0.000292
Pstk	9.30797	11.30899	2.583259	0.000102
Trp53i11	10.33729	11.56382	2.581787	6.85E-07
Zc3h12d	5.14177	6.378172	2.566703	9.44E-07
Kcnn3	13.68957	14.86082	2.559351	4.08E-06

Adtrp	8.650125	9.650504	2.550908	7.80E-06
Iffo2	8.516622	10.06635	2.542368	0.000335
Lrsam1	9.261687	10.24356	2.538689	0.000117
P2ry13	8.462294	10.0607	2.530084	2.68E-05
Adrb2	8.997806	10.20909	2.522677	0.001073
Lrp4	7.719518	8.160936	2.519235	2.22E-05
Slc16a7	9.043995	9.860775	2.519164	7.19E-06
Mfsd6	7.105638	8.165175	2.510898	6.37E-05
Plxnb2	9.191054	10.60218	2.508897	5.18E-05
Vgll4	8.131165	9.956568	2.506279	6.58E-05
Dennd4a	12.86099	14.25433	2.504548	1.46E-05
Ing2	9.023841	9.866032	2.502837	2.64E-05
Eif4ebp1	8.872971	10.32421	2.500471	0.000114
Crot	7.98326	9.550377	2.492182	0.000194
Ksr1	6.449066	7.933039	2.489041	0.000697
Runx1	13.35484	14.90992	2.488386	5.10E-05
Acss1	11.39736	13.06752	2.483725	5.75E-06
Igsf9	5.474906	6.94799	2.48185	1.44E-05
Ermp1	8.639774	9.522296	2.476505	6.90E-06
Rnf180	11.18835	12.9855	2.470351	1.96E-06
Fes	9.741296	11.36632	2.469559	2.31E-06
Mid1	10.64625	11.92459	2.468844	0.00018
Klf9	6.524963	7.91949	2.468392	1.43E-06
Trim25	12.32937	13.65936	2.464985	2.03E-05
Zfp703	9.433423	10.71225	2.462225	1.84E-06
Usp2	8.562047	10.25983	2.44284	3.65E-05
Grhl1	8.093305	9.385497	2.440851	0.000156
Dok2	10.47042	12.20055	2.423935	3.26E-05
Anln	6.428981	7.257383	2.409144	0.000604
Dusp18	6.278502	7.076735	2.404142	0.000905
Pygo2	10.62473	12.22037	2.402725	0.000203
Atm	7.167257	7.760499	2.400295	0.000157
Ubald1	8.171717	9.425666	2.398784	0.000126
Bcl9l	7.575121	9.057609	2.394984	5.36E-06
Ccnd3	13.27797	15.08496	2.387875	8.35E-06
Sp4	7.389856	8.730085	2.387571	3.29E-05
Ldlrad3	7.226661	8.448489	2.38128	0.001553
Ripk2	7.832139	9.406535	2.378339	3.26E-05
Frmd4b	8.546492	10.38453	2.376448	0.000231
Ptger4	9.79787	10.68101	2.374513	1.28E-05
Spag11b	8.679533	9.050128	2.371491	0.000128

Naprt	6.97663	8.244894	2.37091	1.88E-06
Ypel3	11.97981	13.29955	2.364577	1.53E-06
Bmf	5.578903	6.982881	2.35804	7.44E-06
1810011H11Rik	7.286027	8.623318	2.354731	8.70E-05
Trpv2	14.13138	14.78982	2.346042	0.000191
Spaca6	5.482455	6.473086	2.338618	0.00226
Ehbp1	9.200286	10.49083	2.33451	0.005107
Tespa1	5.422031	7.434008	2.332896	0.007552
Ptprs	10.3664	11.6444	2.327589	0.000262
Siglece	8.565837	9.972027	2.323562	7.06E-06
Il1rl2	8.770673	10.3466	2.32008	9.94E-06
Klf3	8.816967	10.44866	2.316083	0.006555
Cidec	10.28255	11.61483	2.315303	2.51E-06
Tex9	5.751748	6.880046	2.311902	0.000192
1190002N15Rik	8.962348	10.0512	2.311543	1.12E-05
Ppp1r3d	9.167481	10.78537	2.306465	8.45E-05
Epb41l1	6.686812	7.457191	2.304915	3.01E-06
Agpat9	10.35175	11.21873	2.299719	6.60E-05
Cd80	12.33433	13.88641	2.299188	5.44E-05
Pnpla5	6.704049	7.872777	2.282528	1.83E-05
Phf21a	6.57492	7.842217	2.278378	7.29E-06
Slc35d2	6.644688	7.063483	2.27327	0.002022
Fam69a	13.70881	14.7328	2.269126	8.99E-06
Sec24b	9.779734	10.93056	2.263971	2.94E-05
Ski	11.27984	12.63076	2.260184	2.57E-06
Glipr2	9.09538	10.24085	2.253963	6.01E-05
Fam46a	7.379038	8.562982	2.251426	4.11E-06
Rasgrp4	8.532905	9.489658	2.249008	4.08E-06
Pld1	8.605384	9.546354	2.246517	4.89E-06
Rasgef1b	11.51309	12.79459	2.245511	0.000292
Ccdc191	6.584948	8.183446	2.236776	0.0009
Nr3c2	5.456361	6.664677	2.232661	0.01418
E2f1	7.51457	8.340746	2.231472	1.91E-05
Rhoh	7.812484	9.344893	2.230382	0.000255
C2cd5	9.108912	10.14787	2.229022	8.82E-06
Dna2	6.111126	6.962141	2.227132	0.000404
Rasgrf2	6.067828	7.013043	2.226493	8.29E-06
Rara	11.37811	12.78903	2.225561	0.000353
Akr1e1	10.21881	10.97873	2.222417	4.62E-06
Megf9	11.05543	12.2563	2.219003	2.84E-05
Pik3cg	7.636449	8.540086	2.216381	5.75E-06

Gm3435	7.714767	8.431373	2.2162	0.000115
Pald1	11.20365	12.49855	2.215704	2.48E-05
Slc35c2	6.992793	7.685654	2.213826	4.97E-05
Recql	10.12851	11.10065	2.213269	0.000274
Gm10797	5.601772	6.612988	2.213109	0.015479
S100a6	9.60312	10.15225	2.210458	6.85E-05
Prom1	11.14902	12.08052	2.209331	0.000185
Bcar3	11.54263	12.35988	2.208249	0.000571
Trim65	6.207408	7.475823	2.205742	0.000391
Btbd2	7.822829	9.087224	2.204693	0.000128
Klrg2	6.085453	7.181325	2.195714	1.28E-05
Gm4980	7.261935	8.233572	2.192436	0.000206
Nrip1	7.4727	9.238902	2.191861	0.000319
Gpr33	6.650725	7.763475	2.183581	0.000581
Tbrg3	6.446964	6.920818	2.18226	0.000611
Mtmr10	9.313081	9.628961	2.180055	0.000669
Ldoc1l	5.226457	6.394308	2.179422	0.001549
Noct	8.31511	9.289467	2.179374	0.000696
Odf2	7.887866	8.393491	2.17799	0.00141
Slc16a14	5.137078	5.940722	2.177059	0.000139
Acvr1c	4.319005	5.624931	2.176645	0.000707
Gm11099	6.08053	7.536112	2.176306	5.54E-05
Sap25	12.07217	13.2091	2.165102	1.80E-05
St3gal4	8.212519	9.210753	2.164402	0.000228
Tnfrsf18	6.618664	7.86683	2.162508	0.003686
Lpar5	5.823434	7.06099	2.160238	0.000484
Trim5	8.515814	10.00752	2.159973	0.000299
Nicn1	9.134775	10.09865	2.159432	2.62E-05
Gpcpd1	12.18037	13.08557	2.151842	5.98E-05
Msrb2	8.554966	9.452445	2.148445	0.000146
D630039A03Rik	6.585991	7.84779	2.141334	0.000157
Olfr115	6.383395	6.133387	2.1396	0.004478
Sgpp1	9.392818	10.36742	2.138046	9.66E-05
Ppp1r21	9.799597	11.03056	2.136444	3.01E-05
Trim24	8.086839	9.270508	2.136137	3.33E-05
L1cam	13.28272	13.86411	2.136133	0.000115
Cdkl5	6.044988	7.446248	2.134648	8.38E-05
Gm10337	4.492491	5.264138	2.134166	0.011313
Nbeal2	5.305772	5.942543	2.133625	0.000317
Trim14	4.871039	5.91389	2.131942	9.10E-05
Dock8	11.77152	12.79178	2.131132	9.44E-06

Adamts6	5.301492	6.098682	2.130795	0.009364
Fgf1	5.488654	6.608682	2.130558	0.00487
Gm21833	10.28263	10.72926	2.130486	0.006345
Golm1	10.57984	11.64686	2.130418	2.98E-06
Kank3	8.556131	9.918628	2.128444	0.000351
Kcnk6	10.32564	11.58807	2.126908	0.002628
Dtna	9.834495	10.72944	2.126409	0.008358
BC052040	7.533652	8.487226	2.123851	0.000442
B4galnt1	13.34305	14.41262	2.120503	4.75E-06
Gmcl1	8.598476	9.903566	2.116423	0.012256
Fam196b	7.137631	7.777332	2.11222	0.000346
Hist1h1c	12.19765	13.61257	2.108826	1.57E-05
Mcm9	6.617261	7.103915	2.107762	0.003362
Carhsp1	12.68771	13.99103	2.105234	1.04E-05
Nab2	8.917065	9.870398	2.103581	0.000121
C2cd2l	12.27467	12.90512	2.102575	1.39E-05
Hepacam2	7.686646	9.334016	2.100979	0.000301
Ddx26b	9.680249	10.34251	2.098684	7.62E-05
Vstm2a	12.10852	12.90772	2.098101	1.83E-05
Xrcc6bp1	5.339983	5.730773	2.09492	0.000565
Iffo1	7.250968	8.467712	2.091874	5.23E-05
Mtap7d3	8.454048	9.81267	2.083803	5.99E-05
Dysf	7.544642	8.024613	2.083612	0.001502
Lpar4	6.663051	8.027288	2.081698	0.001634
Bmx	6.877043	7.533252	2.078536	0.000479
Mfap3l	6.316378	7.292891	2.077809	0.000857
Plcl2	8.96652	10.10609	2.077683	1.58E-05
Csrnp1	7.205327	8.644223	2.070668	0.002325
Cd180	8.253455	9.01892	2.0673	0.003717
Tmcc3	10.84715	11.65397	2.066367	7.23E-06
Batf	10.24537	11.51189	2.066126	0.000452
Trim8	11.1923	12.82002	2.066116	1.82E-05
Sos2	9.426125	10.2247	2.064793	0.001826
Fam212a	5.363164	6.404734	2.063146	0.019292
Peli2	7.560109	8.901441	2.060722	0.000567
Ctc1	6.109008	7.11382	2.060018	0.000442
Mef2c	6.097576	6.706593	2.059408	0.000624
Dcakd	8.844824	10.14553	2.057745	0.000211
Il1r1	5.054068	5.856403	2.054633	0.021273
Lipf	12.06877	12.72382	2.054042	5.76E-05
Phxr1	6.004583	6.445766	2.053718	0.002577

Socs2	7.359234	9.032832	2.050899	0.001563
Klf8	7.101332	8.268849	2.049381	0.000228
Scml4	5.360331	5.727706	2.049277	0.000266
E2f2	8.086977	9.088102	2.047209	0.000568
Slc41a1	12.11848	13.39853	2.045353	0.000424
Calcrl	9.431299	10.18072	2.043049	0.000291
Clmn	11.58506	12.40755	2.035649	3.01E-05
Ndrg1	6.778299	7.683719	2.034435	8.47E-05
Phka2	7.468888	8.435245	2.032316	1.13E-05
Ttc13	9.198283	9.752033	2.029763	0.000603
Pdlim2	7.557143	9.024	2.029728	0.001052
Vamp1	7.569896	8.3566	2.029472	0.002055
Ahnak	10.11502	10.46827	2.028624	0.000238
Itpripl1	9.042672	10.01874	2.021447	0.000221
9430069I07Rik	8.066917	9.164215	2.02137	8.04E-05
Tmem150b	7.284261	7.720932	2.021078	0.001395
Apbb2	8.969118	9.240625	2.01921	0.011805
Ptger1	9.294866	9.961028	2.0182	0.000118
Ogfrl1	8.136902	9.236396	2.015867	0.000296
Rfx2	4.599145	5.515968	2.014845	0.006706
Fam120c	10.5471	11.44048	2.014534	0.000253
Mum1	9.189877	10.08366	2.013286	5.94E-05
Tgfb2	7.827892	8.73205	2.011995	6.15E-05
9330151L19Rik	7.997434	8.893455	2.010367	6.57E-05
Gm17364	5.385416	6.311304	2.005546	0.000826
Cdc6	6.752983	7.662511	2.002457	7.82E-06
Ptpn1	15.21932	14.31325	-2.00109	7.01E-06
Cxcr2	8.057462	6.970156	-2.00848	3.70E-05
Ogfr	9.001121	8.410512	-2.01911	0.000764
Xlr5c	4.334323	4.120884	-2.01912	0.000738
Abhd17c	8.845442	7.793863	-2.02521	0.000832
Retnla	4.72645	3.914103	-2.02635	0.000365
Nlrp3	10.53378	9.583594	-2.02746	0.000134
Pla2g7	6.797397	5.211314	-2.02871	0.006854
Mmp13	6.331057	5.21682	-2.02925	0.021673
Mmadhc	11.44708	11.06433	-2.03068	0.000765
Irf7	4.626122	4.153957	-2.03107	0.000202
Mllt6	5.338707	4.175273	-2.03441	0.000256
Tmprss7	5.752381	5.01825	-2.0368	0.000166
Gch1	14.25129	13.25784	-2.03886	5.72E-06
Fyb	5.127109	4.661483	-2.03943	0.001254

Tefm	9.746311	8.748748	-2.03978	0.000223
Igsf6	11.63771	10.4894	-2.0422	0.000435
Slfn3	7.218856	6.184657	-2.04429	0.000631
Aldh1a1	4.5436	4.118259	-2.04536	0.003097
Cdkn2b	9.192964	7.901735	-2.04693	1.52E-05
BC051142	5.166753	4.833363	-2.0471	0.002504
Sfmbt1	8.02363	6.944452	-2.05397	0.000153
Sqstm1	16.69328	15.47855	-2.05454	2.04E-05
Rnf149	12.51457	11.40649	-2.05632	1.74E-05
Slc37a2	11.83545	10.64123	-2.05692	0.000795
Ccr9	4.902649	4.134927	-2.05912	0.00062
Mical2	10.35144	9.111458	-2.06059	0.000587
Krt222	5.230733	4.554972	-2.06201	0.000209
Rufy3	8.934327	7.740218	-2.06235	0.000983
Nfkbiz	9.663326	9.046274	-2.06278	0.00246
Ikbke	7.942998	7.498886	-2.06456	0.004572
Tnip1	10.3401	9.427458	-2.06619	0.001333
Tubb2a	12.27098	10.18476	-2.06701	0.001483
Xdh	9.618112	8.847397	-2.07202	6.49E-05
Rab3il1	10.12381	9.293323	-2.07476	2.44E-05
Gzma	4.148436	3.890149	-2.07628	0.000312
Gla	10.01505	8.398751	-2.0774	0.007263
Cadm1	5.834994	5.672493	-2.07866	0.011242
Ric1	13.22627	12.23317	-2.08005	2.06E-05
Prune	12.12416	10.61891	-2.08556	0.000251
Il12b	6.78647	6.790125	-2.0933	0.0003
Ppfia3	7.29301	6.387059	-2.09421	0.000936
Gm21836	6.661425	6.070705	-2.09819	0.008889
Isg15	6.082044	5.515215	-2.10105	0.000743
B4galt5	11.90821	10.52887	-2.10494	0.001365
Serpinb6b	6.142219	5.791186	-2.10541	0.000915
Nr1h3	13.49208	12.37814	-2.10731	4.06E-06
Osbpl8	15.23923	13.88546	-2.11088	0.000133
Pim1	11.72384	10.50885	-2.11433	0.011406
Sftpa1	6.192892	5.866783	-2.11463	0.000461
Gde1	13.25801	11.97193	-2.1157	0.000307
Casp4	12.5183	12.14053	-2.11777	5.19E-05
Fabp4	16.68024	15.48515	-2.11781	3.41E-05
Gramd1a	11.84074	10.73131	-2.12176	2.01E-05
Daam1	11.81834	10.35443	-2.12412	2.86E-05
Jade3	8.831844	7.07099	-2.12927	5.10E-06

Smpdl3b	11.64864	11.34258	-2.13302	0.000107
Gypa	4.286162	3.99061	-2.15443	0.007111
Abcb1b	7.302398	6.302436	-2.15461	0.000239
Atp6v1a	11.67534	10.04457	-2.15618	3.56E-05
Mkl1	10.4763	9.437487	-2.15623	3.23E-05
Fmn1	10.29003	8.736487	-2.1574	0.000293
H2-Q1	6.607408	6.009896	-2.16411	4.82E-05
Tpra1	9.224939	8.052018	-2.16496	7.49E-05
Slc31a1	11.51195	10.29009	-2.17013	1.08E-05
Muc1	6.434777	6.5253	-2.17403	0.000338
Mov10	8.04127	7.317968	-2.1742	0.001256
Crem	10.4755	9.868263	-2.17422	0.004867
Relb	7.160769	6.111238	-2.185	0.000355
Fzd8	8.039509	6.543053	-2.19152	1.61E-05
Sftpc	10.92692	10.45186	-2.19241	0.017779
Rnf19b	11.45001	10.48637	-2.19595	1.15E-05
Raf1	13.85991	12.37616	-2.19738	4.18E-06
Ablim1	4.835122	3.507963	-2.21057	0.000102
Cacul1	11.87117	10.5128	-2.21652	2.73E-06
Slfn8	7.231488	6.166419	-2.21937	0.003438
Rit1	13.48311	12.33994	-2.22008	5.89E-05
Malt1	12.80743	11.53043	-2.22044	5.90E-05
Cd274	13.81532	12.81703	-2.22046	1.28E-05
Mmp12	15.81282	13.95149	-2.22397	0.001819
Ccdc130	10.64317	9.53276	-2.22459	9.08E-05
Atad5	5.580834	5.077637	-2.22582	0.002316
Slamf8	4.119643	3.837375	-2.23607	3.97E-05
Bhlhe41	11.62786	10.20146	-2.23616	2.90E-05
Irgm1	6.887709	6.261588	-2.2444	9.99E-06
N4bp2l1	7.807985	7.223438	-2.24705	0.000506
3110043O21Rik	11.73214	10.26219	-2.24814	0.000152
Myo1e	12.88052	11.7751	-2.24909	1.62E-05
Hsd11b1	10.70707	9.845127	-2.26334	9.29E-05
Gm21860	10.65596	7.719332	-2.27028	0.049609
Gm21748	10.65596	7.719332	-2.27028	0.049609
Sccpdh	9.180801	8.096419	-2.2742	0.000569
Tspan5	11.5218	10.34624	-2.28132	0.00018
Zfp143	11.33741	9.92376	-2.28332	7.27E-06
Plekho1	11.06048	10.15172	-2.28434	0.000165
H2-M2	7.247414	6.257611	-2.28677	1.67E-05
Fam26f	6.337433	6.821781	-2.28929	0.000271

Ubap1	15.2891	14.09584	-2.28979	0.000225
Cdc7	7.834194	6.709748	-2.29282	0.001354
Lif	8.467676	7.100577	-2.29357	0.003306
Ttc1	9.958118	8.818646	-2.30827	1.50E-05
Dnmt3a	9.563043	7.976544	-2.31065	2.20E-05
Fdps	12.33601	11.43962	-2.31581	0.003379
Gdap10	11.55866	9.60647	-2.32545	0.027261
Sik3	12.7544	11.39164	-2.32671	1.54E-06
Anpep	11.75555	9.40099	-2.32934	0.006809
Got1	10.52418	9.685629	-2.34387	3.34E-05
Gdpd1	10.13495	8.847831	-2.34543	0.000809
Cd300lb	10.1488	8.737816	-2.34676	1.14E-05
Eaf1	13.31525	11.43663	-2.37081	1.73E-05
Clec4d	15.14104	13.60917	-2.3793	5.20E-05
Fam49a	10.16002	9.649895	-2.38347	0.000132
Tnfrsf1b	12.839	11.28367	-2.38863	9.20E-06
Cxcl2	12.89397	11.45635	-2.39357	0.002802
Phlpp1	11.20519	10.04439	-2.39391	4.68E-05
Rtp4	6.450633	5.99132	-2.39485	0.001547
Clu	7.713774	7.086148	-2.3952	0.000618
Treml4	12.63606	10.91512	-2.40004	0.000441
1110007C09Rik	10.93228	9.416231	-2.40119	2.51E-05
AA467197	9.703311	9.345062	-2.40139	0.000144
Tmem51	10.38873	9.337306	-2.40975	1.95E-05
Icosl	10.65761	9.44515	-2.41993	7.24E-06
Tm4sf1	5.093132	4.517742	-2.4272	0.000226
Tapbpl	10.17571	9.653385	-2.43224	1.09E-06
Fas	7.299046	6.24804	-2.43713	2.11E-05
Gm12185	5.052191	4.741407	-2.43868	0.000927
Ifitm3	7.2222	6.775506	-2.44114	0.000151
Plbd1	8.412211	8.385631	-2.44892	0.000826
Clcf1	10.29434	8.178775	-2.44994	0.001471
Clec4e	16.43962	15.07191	-2.45444	1.94E-05
Arg2	7.267277	5.805379	-2.46067	7.51E-06
Slc11a1	11.09123	8.600148	-2.46133	0.002189
Trim13	8.834805	7.138067	-2.46136	6.33E-06
Gbp2	5.829648	6.512268	-2.46426	5.82E-05
Tatdn2	8.066849	6.850703	-2.47235	0.000111
Gpr65	7.075792	6.819673	-2.47341	9.91E-06
Plekhg1	15.10742	13.41087	-2.48673	0.000348
Htra4	9.253272	8.315264	-2.4903	9.08E-05

Mnda	9.411069	8.843709	-2.49075	1.40E-05
Stra6l	10.28262	8.154396	-2.50114	0.000103
Serpinb9	6.567934	5.611269	-2.51588	4.62E-05
Cblb	14.40328	13.43027	-2.51607	2.88E-06
Wdr91	14.84825	13.08488	-2.52179	5.70E-06
Ptges	10.67426	8.91199	-2.5392	0.004317
Rgl1	9.541856	7.858815	-2.5426	0.000137
Traf3	7.930715	6.49966	-2.54506	2.33E-05
Slc11a2	8.746022	7.32836	-2.56758	5.65E-05
Cbwd1	10.25183	8.889605	-2.57961	3.06E-06
Smim22	6.971025	6.597888	-2.58149	0.0001
Akr1b8	12.3622	9.983504	-2.59192	0.000445
Rab20	8.694111	7.673419	-2.62683	4.74E-06
Mpp7	10.92101	9.723267	-2.62781	0.000702
Top1	13.59142	11.98028	-2.64031	9.07E-06
Spryd7	13.17822	11.44275	-2.6486	1.02E-05
Lap3	9.119898	8.73098	-2.67345	0.000676
Spata13	8.103891	7.611706	-2.6738	2.57E-05
Ppard	12.78765	10.26847	-2.68784	1.29E-05
Fosl1	8.438531	6.45405	-2.7043	0.000134
Ptprj	14.3433	12.85467	-2.70864	7.14E-06
Nrp2	14.47881	12.95903	-2.7368	1.58E-05
Dcstamp	9.462183	8.786114	-2.73968	6.00E-05
Pmp22	9.431987	7.301046	-2.74053	0.000161
Rgs1	13.58022	12.12157	-2.74624	3.18E-05
Capn15	10.85849	9.144101	-2.7494	1.83E-06
Mtmr14	11.38672	9.998529	-2.75415	1.10E-06
Cd14	13.84661	12.83895	-2.75705	8.70E-06
Klrd1	6.409701	6.029455	-2.7667	1.21E-05
Plau	10.05588	8.529654	-2.80414	5.24E-05
Mitf	12.35036	10.96902	-2.80526	3.66E-06
Cldn18	7.575062	7.076032	-2.82112	0.00116
Pigr	4.854699	4.5275	-2.82395	0.001179
Vps37b	6.192707	4.466415	-2.83446	9.76E-06
Ccl22	9.310214	7.868044	-2.8369	1.21E-06
H2-Q4	6.210775	5.864084	-2.86316	2.87E-06
Esd	14.61871	12.70303	-2.87345	8.73E-07
Sod2	12.00371	10.60894	-2.88502	9.29E-07
Abcb1a	9.591409	8.216024	-2.90408	0.001576
Cd3d	5.717498	4.977509	-2.9276	4.01E-06
Smox	12.69175	11.67702	-2.94606	4.39E-06

Plxna1	11.99332	9.863512	-2.95758	1.04E-05
Ly6c2	4.339217	4.054254	-2.96928	0.000167
Flrt3	11.43818	9.643953	-2.97339	0.000255
Gpr132	6.676692	5.893618	-2.97486	1.28E-05
Por	15.01553	12.86903	-2.99278	2.24E-06
Stxbp1	9.060698	8.034779	-3.00399	8.32E-06
Nol4l	14.04522	12.35103	-3.00532	2.30E-07
Etv3	11.31174	9.560156	-3.01625	2.40E-05
Ces1d	6.474257	5.846605	-3.02179	0.000156
Nod2	6.698669	6.179768	-3.02415	4.45E-05
Abcc1	10.71743	8.303565	-3.02967	1.87E-05
Lrp8	10.95637	8.180014	-3.04276	4.99E-05
Txnrd1	16.73497	14.81561	-3.06512	2.28E-06
Tgif1	13.70569	12.13594	-3.12651	8.41E-07
Bcl2l1	11.84858	10.28661	-3.16632	1.95E-05
Nlrc5	5.113567	5.088975	-3.17551	8.41E-07
Gclm	14.41264	12.28916	-3.21298	5.90E-06
Reg3g	5.8409	5.108737	-3.23073	2.19E-07
Dusp10	10.76101	8.357567	-3.26682	0.000661
Igf2bp2	11.0263	8.855131	-3.34688	7.40E-06
Slc40a1	9.163085	6.395398	-3.35263	1.53E-05
Tns3	8.997355	7.79706	-3.37579	6.03E-05
Gdf15	11.71527	9.51415	-3.38481	2.27E-05
Nfkb2	14.33134	12.52142	-3.40621	5.14E-07
Tshz1	11.54069	9.544508	-3.43685	3.39E-06
Tma16	11.84815	9.929958	-3.44012	3.27E-06
Tor3a	12.67201	10.56014	-3.44232	2.10E-07
Zswim4	13.40424	11.77694	-3.46613	2.17E-07
Mreg	9.354104	7.00227	-3.49039	6.34E-07
Gas2l3	12.00222	9.926944	-3.49196	7.00E-07
Ly6c1	4.649626	4.767731	-3.49804	5.97E-06
Zc3h12c	7.026305	5.155932	-3.51506	0.001783
Plac8	4.191997	3.883665	-3.66318	0.001636
Dnmt3l	7.902083	5.173162	-3.67667	8.03E-07
8030474K03Rik	5.036066	4.88648	-3.69766	0.006807
Fam20c	6.920183	5.652619	-3.699	2.52E-07
Pde4b	13.19683	11.93431	-3.74216	5.24E-05
Cyfip2	9.995325	7.209837	-3.76216	0.000507
Gclc	13.02114	10.52396	-3.76859	1.27E-06
Tbc1d10a	8.846422	7.092433	-3.791	4.28E-07
Ugcg	12.7596	10.91292	-3.91757	4.05E-07

Ninj1	13.99865	12.08586	-3.92517	1.81E-06
Zeb2	13.34707	11.21831	-4.04565	4.19E-07
Nfkbie	8.205057	6.13467	-4.05988	4.51E-06
Depdc7	5.406562	3.582363	-4.09522	5.23E-08
Slamf7	8.746058	6.979742	-4.10789	4.03E-06
Cyth3	13.25219	11.27026	-4.11161	9.05E-09
H2-Q8	8.889223	8.36659	-4.17898	9.97E-05
Ext1	13.21447	10.76633	-4.26538	1.65E-06
Cd200	8.952255	6.260718	-4.31142	4.07E-07
Nfkb1	14.02112	11.93486	-4.31451	3.38E-07
Ch25h	13.23465	10.50346	-4.31467	0.000137
Bcl3	9.14023	7.442856	-4.32436	2.00E-07
Icam1	14.97747	13.03066	-4.37412	1.05E-07
Ptafr	12.58699	10.73596	-4.40846	2.41E-06
Lad1	8.930443	6.937136	-4.42836	3.40E-06
Gadd45a	9.202176	7.077454	-4.45195	1.93E-06
Optn	8.900141	6.200272	-4.62447	8.47E-06
Fnip2	9.109412	6.628	-4.63135	8.20E-06
Ramp3	8.966291	7.39123	-4.68068	2.02E-05
Ocstamp	6.766606	4.604444	-4.73047	5.08E-06
Inhba	8.391508	5.548024	-4.79208	0.000298
Rbm47	9.441772	6.895131	-4.86593	3.32E-06
Cxcl9	5.412461	5.843822	-4.88867	3.80E-06
Fyn	11.49244	9.381921	-4.90049	2.33E-08
Ptgir	10.43227	7.093151	-4.91552	2.94E-06
Ltf	5.743214	4.6564	-5.05756	5.27E-06
Nampt	12.62931	10.0455	-5.06188	1.55E-08
Cxcl16	13.30489	11.62905	-5.22423	8.29E-09
Ctla2a	6.990299	6.525865	-5.36877	2.66E-07
Adam8	6.687077	4.540859	-5.3702	1.97E-08
Pik3r5	13.23677	10.52385	-5.53656	3.23E-09
Tnfaip3	13.88723	11.47418	-5.55658	1.10E-05
H2-Q7	8.114265	7.614184	-5.91947	6.25E-07
Il2rb	4.959077	4.393296	-6.22487	1.65E-07
Cxcr1	13.44706	9.965301	-6.30412	1.77E-05
Cyp2f2	7.441815	6.263609	-6.31907	1.47E-07
H2-Q5	9.299653	8.542645	-6.41155	4.33E-06
Bcl6	9.693866	6.881182	-6.47443	6.06E-06
Ass1	9.787403	7.02185	-6.74642	1.27E-05
Srxn1	14.89214	11.48677	-7.05288	1.28E-07
Met	8.883626	5.234829	-7.43127	3.07E-06

Snca	4.84575	4.765065	-7.93469	0.04159
Hmox1	15.57168	11.63369	-9.27092	2.78E-07
Cbr2	8.222673	6.748054	-10.3622	9.67E-09
Irg1	7.805917	5.058332	-11.3328	0.000135
Scgb1a1	8.92432	7.480078	-12.7985	1.81E-08
Slc7a11	15.34694	11.04814	-13.3992	5.70E-05
Scgb3a1	8.297481	7.168629	-13.711	1.32E-08
Hbb-bt	13.99633	13.81878	-14.3888	0.038516
Cyp2a5	7.141375	5.798252	-14.5425	1.02E-05
Hbb-bs	14.12558	13.82157	-15.2141	0.036291
Tspan1	6.056071	4.582445	-17.9147	1.08E-07
Alas2	6.084054	6.247352	-18.5512	0.01555
Scgb3a2	10.80422	8.917808	-20.4658	8.10E-07
Bpifa1	9.807678	7.959314	-21.0855	4.30E-08
Hba-a1	12.2778	12.12577	-29.0275	0.028137
Hba-a2	12.14128	12.0835	-32.4278	0.025885
Bpifb1	6.784819	4.860209	-33.0408	2.40E-07
Plk2	12.24066	6.17439	-39.3205	3.82E-09
Plpp3	14.08884	7.657022	-44.8164	7.22E-12

Table S10: Primary metabolic process genes upregulated in alveolar macrophages from MAS resolution

Gene symbol	Gene description	Log change (MAS resolution vs control)	P value
Il6ra	interleukin 6 receptor, alpha	2.95296	2.51E-06
Id1	inhibitor of DNA binding 1	2.715936	2.76E-08
Per2	period circadian clock 2	2.622686	9.00E-08
Cbx4	chromobox 4	2.578772	2.17E-07
Trerf1	transcriptional regulating factor 1	2.571757	1.99E-08
Nr4a2	nuclear receptor subfamily 4, group A, member 2	2.511314	1.08E-06
Klf13	Kruppel-like factor 13	2.369845	2.10E-07
Rgs2	regulator of G-protein signaling 2	2.238358	4.34E-07
Tsc22d3	TSC22 domain family, member 3	2.163371	1.85E-07
Abcd2	ATP-binding cassette, sub-family D (ALD), member 2	2.160568	1.45E-06
Nr4a1	nuclear receptor subfamily 4, group A, member 1	2.09268	2.09E-06
Dusp7	dual specificity phosphatase 7	2.080029	2.73E-07
Angptl4	angiopoietin-like 4	2.069016	3.37E-08
Fzd4	frizzled homolog 4 (Drosophila)	2.058217	4.40E-07
Klf4	Kruppel-like factor 4 (gut)	2.033787	1.78E-08
Pstk	phosphoseryl-tRNA kinase	2.001017	3.01E-05
Zfp36l2	zinc finger protein 36, C3H type-like 2	1.996971	7.94E-08
Txnip	thioredoxin interacting protein	1.96094	9.23E-07
Jarid2	jumonji, AT rich interactive domain 2	1.956121	3.40E-08
Serpine1	serine peptidase inhibitor, clade E, member 1	1.849985	5.01E-06
Erf	Ets2 repressor factor	1.832573	1.31E-06
Tet2	tet methylcytosine dioxygenase 2	1.826593	3.02E-06
Vgll4	vestigial like 4	1.825403	1.21E-06
Ccnd3	cyclin D3	1.806985	2.65E-07
Rnf180	ring finger protein 180	1.79715	8.47E-08
Nrip1	nuclear receptor interacting protein 1	1.766203	9.69E-06
Tnfsf18	tumor necrosis factor (ligand) superfamily, member 18	1.760929	0.001274
Rassf5	Ras association (RalGDS/AF-6) domain family member 5	1.744688	1.40E-07
Pdgfc	platelet-derived growth factor, C polypeptide	1.735633	1.92E-07
Agfg2	ArfGAP with FG repeats 2	1.726141	1.80E-06
Tlr3	toll-like receptor 3	1.709315	1.46E-05
Usp2	ubiquitin specific peptidase 2	1.697788	1.16E-08
Klf3	Kruppel-like factor 3 (basic)	1.631693	0.000173
Ppp1r3d	protein phosphatase 1, regulatory subunit 3D	1.617894	2.82E-08
Pygo2	pygopus 2	1.595643	1.88E-06
Ripk2	receptor (TNFRSF)-interacting serine-threonine kinase 2	1.574396	6.76E-06
Runx1	runt related transcription factor 1	1.555088	2.43E-06
Cd80	CD80 antigen	1.552073	3.07E-06
Cnksr3	Cnksr family member 3	1.52654	9.71E-06
Btg2	B cell translocation gene 2, anti-proliferative	1.50551	0.000189

Table S11: Quantitative PCR primers used in this study.

qPCR Method	Target Name	Primer Sequence	Supplier
SYBR Green	GAPDH	Forward: AAGGTCGGTGTGAACGGATTT<br>Reverse: AATTTGCCGTGAGTGGAGTCATAC	Integrated DNA Technologies
	CXCL9	Forward: CACGATCCACTACAAATCCC<br>Reverse: TTCACATTTGCCGAGTCC	
	TGFß	Forward: GCGGACTACTATGCTAAAGAGG<br>Reverse: GTAGAGTTCCACATGTTGCTCC	
	HO-1	Forward: GTGACAGAAGAGGCTAAGACCG<br>Reverse: ACAGGAAGCTGAGAGTGAGGAC	
	KLF13	Forward: ACACTCTTGCATTACCCACTCTT<br>Reverse: CCTGGTTTCTATTCCTGCTGTC	
	IL-12	Forward: AAGACGTTTATGTTGTAGAGGTGG<br>Reverse: ACTGGCCAGTATCTAGAAACTCTTT	
	IL-10	Forward: GACAATAACTGCACCCACTT<br>Reverse: TCAAATGCTCCTTGATTTCT	
TaqMan	sno135	CTAAAATAGCTGGAATTACCGGCAGATTGGTAG<br>TGGTGAGCCTATGGTTTTCTGAAG	ThermoFisher
	hsa-miR-146a	UGAGAACUGAAUUCCAUGGGUU	
	hsa-miR-125a-5p	UCCCUGAGACCCUUUAACCUGUGA	
